# The antennal transcriptome of *Triatoma infestans* reveals substantial expression changes triggered by a blood meal

**DOI:** 10.1186/s12864-022-09059-6

**Published:** 2022-12-30

**Authors:** Jose Manuel Latorre Estivalis, Lucila Traverso, Gina Pontes, Marcelo Gustavo Lorenzo

**Affiliations:** 1 Laboratorio de Insectos Sociales, Instituto de Fisiología, Biología Molecular y Neurociencias, Universidad de Buenos Aires - CONICET, Ciudad Autónoma de Buenos Aires, Argentina; 2 Laboratorio de Neurobiología de Insectos (LNI), Centro Regional de Estudios Genómicos, Facultad de Ciencias Exactas, Universidad Nacional de La Plata, CENEXA, CONICET, La Plata, Buenos Aires, Argentina; 3 Laboratorio de Eco-Fisiología de Insectos del Instituto de Biodiversidad y Biología Experimental y Aplicada (IBBEA-UBA-CONICET), DBBE - Facultad de Ciencias Exactas y Naturales, Universidad de Buenos Aires, Ciudad Autónoma de Buenos Aires, Argentina; 4 Vector Behaviour and Pathogen Interaction Group, Instituto René Rachou - FIOCRUZ-Minas, Belo Horizonte, Minas Gerais, Brazil

**Keywords:** *Triatoma infestans*, Heteropterans, Sensory genes, Transcriptome, Host-seeking, Blood meal

## Abstract

**Background:**

*Triatoma infestans* is the main vector of Chagas disease in the Americas, currently transmitting it in Argentina, Paraguay, and Bolivia. Many *T. infestans* populations present insecticide resistance, reducing the efficiency of control campaigns. Alternative vector control methods are needed, and molecular targets mediating fundamental physiological processes can be a promising option to manipulate kissing bug behavior. Therefore, it is necessary to characterize the main sensory targets, as well as to determine whether they are modulated by physiological factors. In order to identify gene candidates potentially mediating host cue detection, the antennal transcripts of *T. infestans *fifth instar larvae were sequenced and assembled. Besides, we evaluated whether a blood meal had an effect on transcriptional profiles, as responsiveness to host-emitted sensory cues depends on bug starvation.

**Results:**

The sensory-related gene families of *T. infestans* were annotated (127 odorant receptors, 38 ionotropic receptors, 11 gustatory receptors, 41 odorant binding proteins, and 25 chemosensory proteins, among others) and compared to those of several other hemipterans, including four triatomine species. Several triatomine-specific lineages representing sensory adaptations developed through the evolution of these blood-feeding heteropterans were identified. As well, we report here various conserved sensory gene orthogroups shared by heteropterans. The absence of the thermosensor *pyrexia*, of *pickpocket* receptor subfamilies IV and VII, together with clearly expanded* takeout *repertoires, are revealed features of the molecular bases of heteropteran antennal physiology. Finally, out of 2,122 genes whose antennal expression was significantly altered by the ingestion of a blood meal, a set of 41 *T. infestans *sensory-related genes (9 up-regulated; 32 down-regulated) was detected.

**Conclusions:**

We propose that the set of genes presenting nutritionally-triggered modulation on their expression represent candidates to mediate triatomine host-seeking behavior. Besides, the triatomine-specific gene lineages found represent molecular adaptations to their risky natural history that involves stealing blood from an enormously diverse set of vertebrates. Heteropteran gene orthogroups identified may represent unknown features of the sensory specificities of this largest group of hemipteroids. Our work is the first molecular characterization of the peripheral modulation of sensory processes in a non-dipteran vector of human disease.

**Supplementary Information:**

The online version contains supplementary material available at 10.1186/s12864-022-09059-6.

## Background

Understanding the biology of *Triatoma infestans* is highly relevant because it is the main insect vector of the parasite causing Chagas disease in humans [1]. This deadly disease is caused by *Trypanosoma cruzi *and affects millions of people in the Americas [2]. As these authors indicate, the transmission of* T. cruzi *happens fundamentally inside poor rural houses due to the proliferation of bug colonies inside wall cracks which can eventually become infected with this parasite [2]. It is estimated that 13% of the population in Latin America is exposed to vectorial transmission of *T. cruzi* [1] and its control relies heavily on eliminating vector insects from human habitations [3]. Consequently, effective control tools targeting triatomine bugs are permanently needed and this is especially true for the main South American vector species that have developed relevant levels of insecticide resistance [3]. Aiming at specific molecular targets mediating key physiological processes can be considered an alternative for developing rational control tools having less impact on non-target species and the environment. Among these, we suggest that key sensory targets would be specially suitable to interfere bug behavior.

Olfaction is critical for insect survival [4], as it mediates the detection of food [5, 6], sexual partners [7–9], danger [10, 11] and pathogens [12, 13], among other resources and threats. Insects have developed a diverse array of receptors to detect both host cues and communication signals [14]. The main groups of insect proteins mediating the detection of volatile molecules are odorant receptors (ORs), ionotropic receptors (IRs), odorant-binding proteins (OBPs), chemosensory proteins (CSPs), odorant-degrading enzymes (ODEs), and sensory neuron membrane proteins (SNMPs) [15]. The genes coding for membrane receptors mediating odor recognition (ORs and IRs) are expressed by olfactory sensory neurons (OSNs), which are housed in the olfactory sensilla mostly located on insect antennae. This confers these neurons the ability to detect minute quantities of volatile compounds circulating in the environment, granting that brain centers dealing with olfactory information are updated on their presence, abundance, and fluctuations. Nevertheless, this ability is not a static feature, but one that varies with the season, time of the day, development, nutritional and mating status [16]. The modulation of sensory neuron responsiveness seems to be mediated by a variety of correlated changes in gene expression as an underlying molecular substrate [17–22]. Characterizing such molecular changes seems critical to understanding sensory processes and uncovering key components that may become targets for controlling insect pests more rationally. Other genes coding for sensory components that detect mechanical stimuli, heat, humidity, ammonia or salinity are also fundamental to grant insect survival. Gene families like those of transient receptor potential (TRP) channels, *pickpockets *(PPKs) and ammonium transporters (AmTs) represent the molecular substrate underlying these sensory abilities. Detecting heat and humidity seems to play a key sensory role in host recognition by triatomines and mosquitoes [23–26].

In recent years, the advent of next-generation sequencing techniques has allowed the characterization of the sensory gene repertoires of many insects through genomic and transcriptomic studies [27]. The resulting datasets have improved our knowledge about the molecular processes that underlie the behavioral and sensory plasticity of these animals. For example, the characterization of antennal gene expression changes triggered by blood ingestion allowed a better understanding of the molecular bases of host-seeking behavior at the peripheral level in several mosquitoes [17, 20–22]. Even though *T. infestans* is the main vector species transmitting Chagas disease, its genome sequence annotation is not available, hindering homology-based searches of molecular targets. Lacking such a massive source of gene sequences limits our characterization of gene families that have suffered expansions or contractions through the evolution of the species. Besides, it limits our capacity to uncover key genetic components deserving functional studies. Furthermore, this impedes evaluating whether the recent evolutionary pressures caused by bug domiciliation have had any impacts on its genetic sensory machinery.

Few transcriptomic studies have been published for* T. infestans* [28–31], however, none was performed on sensory tissues. In this work, we sequenced the antennal transcripts of *T. infestans* fifth instar larvae, generated a de novo assembly, and studied the effect of blood ingestion on transcriptional profiles in this tissue. The sensory gene repertoire of *T. infestans* was annotated and compared to that of *Rhodnius prolixus* and other hemipterans. This process revealed gene lineages unique to triatomines. Besides, we identified a set of sensory-related genes that had antennal expression levels significantly altered after feeding, which suggests that they are candidates to mediate host-seeking behavior in triatomines. This work represents the first characterization of peripheral modulation of host-seeking in a non-dipteran human disease vector.

## Results

### Sequencing and de novo assembly

More than 688 M paired-end reads of 150 base pair (bp) length (57.3 M reads on average per library) were generated from the sequencing of the 12 antennal samples (Additional file 1: Supplementary Table S1). The assembled transcriptome contained a total of 366,262 transcripts and 261,498,151 assembled bases, with a GC percent of 37. The N50 value of contigs in the assembled transcriptome was 1,253 bp, while the median contig length reached 360 bp and the average contig was 713.96 bp. A total of 78,365 predicted coding sequences (CDSs) were identified and 38,291 of them were maintained after filtering by redundancy. The BUSCO searches based on this dataset revealed 96.4% of completeness (Additional file 2: Supplementary Figure S1).

### Identification of sensory gene families in *T. infestans*

Transcripts from a total of 11 sensory gene families, including different types of receptors and odorant transporters, were identified in our database and annotated according to their phylogenetic relation to *R. prolixus* genes. All *T. infestans* protein sequences and those from other insects used in this work are available in fasta format in Additional file 3: Supplementary Data File S1. The expression values of the sensory genes identified here, represented as Log_10_ (Transcripts *Per* kilobase *per* Million reads—TPM + 1), are detailed in the Additional file 4: Supplementary Table S2.

### Odorant receptors

A total of 127 ORs were identified in the antennal transcriptome, with an average length of 346 amino acids (ranging from 203 to 474) (Additional file 5: Supplementary Table S3). Fifty-six (44%) had their sequences complete, while 71 ORs (66%) presented partial sequences, mainly due to the absence of the initial methionine. All sequences had the PFAM domain PF02949.23 characteristic of the OR family, except for 3 candidates. Besides, 89 sequences (70%) mapped against an insect OR from the UniProtKB/Swiss-Prot database. A total of 95 (75%)* T. infestans *ORs had between 4 and 7 transmembrane domains. The size of the OR repertoire of *T. infestans *(127) is similar to that of* O. fasciatus* (120) and larger than the repertoires found for *R. prolixus* (110) and other hemipterans, such as *Cimex lectularius* (47), *Diaphorina citri* (46) *Tessaratoma papillosa* (59) and *Acyrthosiphon pisum *(79) (Table 1). On the other hand, the OR sets of *Apolygus lucorum *and *Sogatella furcifera* (135), *Halyomorpha halys* (138) and *Nilaparvata lugens* (141) slightly exceed the size of that of *T. infestans* (Table 1).

**Table 1:** The numbers of sensory receptors and odorant carriers in different hemipterans. * genes identified in this study; () numbers reported in previous studies; NA, not available

SUBORDER—INFRAORDER—FAMILY	SPECIES	ORs	GRs	IRs	IR75 members	OBPs	CSPs	TOs	CHEs	TRPs	PPKs	SNMPs	Data origin	Publication
Heteroptera—Pentatomomorpha—Lygaeidae	*Oncopeltus fasciatus*	120	115	37	10	22*	14*	19*	2*	12*	11*	0*	Genome	[32]
Heteroptera—Pentatomomorpha—Pentatomidae	*Halyomorpha halys*	138	198	24	7	44	17	46*	11*	19*	16*	3*	Genome and Transcriptome	[33–35]
Heteroptera—Pentatomomorpha—Tessaratomidae	*Tessaratoma papillosa*	59	NA	14	2	33	NA	NA	NA	NA	NA	NA	Transcriptome	[36]
Heteroptera—Cimicomorpha—Cimicidae	*Cimex lectularius*	47	24	30	12	14	14	11*	0*	13*	10*	2*	Genome	[37]
Heteroptera—Cimicomorpha—Miridae	*Apolygus lucorum*	135	57	33	7	38	8	56*	6*	21*	8*	2*	Genome	[38]
Heteroptera—Cimicomorpha—Reduvidae	*Triatoma infestans*	127*	11*	38*	19*	41*	25	36*	6*	17*	13*	3*	Transcriptome	Present study
Heteroptera—Cimicomorpha—Reduvidae	*Triatoma brasiliensis*	NA	NA	NA	NA	23 (25)	16	19 (22)	NA	NA	NA	NA	Transcriptome	[39]
Heteroptera—Cimicomorpha—Reduvidae	*Triatoma dimidiata*	NA	NA	NA	NA	26 (41)	13 (34)	11*	NA	NA	NA	NA	Transcriptome	[40]
Heteroptera—Cimicomorpha—Reduvidae	*Triatoma pallidipennis*	NA	NA	NA	NA	28 (34)	14 (23)	16*	NA	NA	NA	NA	Transcriptome	[40]
Heteroptera—Cimicomorpha—Reduvidae	*Rhodnius prolixus*	111	28	30	13	27	19	32* (15)	5	14	10	4	Genome	[41–43]
Auchenorrhyncha—Fulgoromorpha—Delphacidae	*Nilaparvata lugens*	141	28	25	0	11	17	NA	NA	NA	NA	NA	Genome	[44, 45]
Auchenorrhyncha—Fulgoromorpha—Delphacidae	*Sogatella furcifera*	135	NA	16	0	12	9	NA	NA	NA	NA	NA	Genome	[45, 46]
Sternorrhyncha—Aphidomorpha—Aphididae	*Acyrthosiphon pisum*	79	77	19	5	18	12	NA	NA	13	21	NA	Genome	[47–49]
Sternorrhyncha—Liviidae	*Diaphorina citri*	46	20	6	0	9	12	NA	NA	NA	NA	NA	Transcriptome	[50]

We were able to annotate 66 out of 127 ORs based on their phylogenetic relations to *R. prolixus* ORs (Additional file 6: Supplementary Figure S2). The phylogenetic tree rooted in the conserved Orco proteins showed that there is a complex relationship between the specific ORs across the six heteropterans here analyzed. As expected, some large species-specific OR expansions were identified, like *OfasOr24*-*59 *from *O. fasciatus*, *HhalOr33-72* from *H. halys*, and a clade from *A. lucorum* composed of *AlucOr13*, *AlucOr15*, and *AlucOr100* among others. Interestingly, *C. lectularius* does not follow this pattern as it has only a few small expansions (with less than 4 members). On the other hand, distinct clades or subfamilies reveal potentially orthologous relationships among heteropteran species, like those that contain *TinfOr1*, *TinfOr52* or *TinfOr105*, and their potential orthologues.

Six clades (highlighted in green in the tree) seem to be exclusive for triatomines: 4 having more than 10 members, and 2 with less than 6 members for each species. In all these clades, *T. infestans* and *R. prolixus* OR numbers seem balanced: 13/12, 7/4, 12/10, 13/13, 30/26, and 8/6. The largest clade among those specific to triatomines included *RproOr58-87* and several* T. infestans* ORs, being the only expanded clade in this section of the tree (Additional file 6: Supplementary Figure S2). A similar pattern could be observed in the adjacent branch, where triatomines also presented an expansion (14 ORs) while the ORs of the other species were less abundant. This part of the tree (branches in dark green) included many triatomine ORs (92), while 65 belonged to the remaining heteropterans. Most of the *T. infestans* ORs were grouped together with *R. prolixus* ORs in clades of varying sizes. In a few cases, triatomine ORs appeared isolated in the tree, such as the *RproOr113-114* clade or *T. infestans* receptors *TRINITY_DN7507_c0_g1_i1.p1* and *TRINITY_DN45340_c0_g1_i2.p1* (Additional file 6: Supplementary Figure S2).

As expected, *TinfOrco* was the OR showing the highest expression in all 12 libraries. Interestingly, *TRINITY_DN2098_c0_g1_i14.p1*, with no orthologue found in *R. prolixus*, also presented a very high expression level in all libraries (Additional file 7: Supplementary Figure S3). The analysis revealed a group of 17 ORs, that included the differentially expressed *TinfOr54* (see Results section "Effect of feeding on antennal transcript abundance"), with high expression within the OR family.

### Ionotropic receptors

A total of 38 IRs and 9 ionotropic glutamate receptors (iGlurs) were identified in our dataset (Additional file 5: Supplementary Table S3). Twenty-one of them (55%) had complete sequences, while the remaining showed partial sequences, mainly due to the absence of the initial methionine. At least half of the *T. infestans* IRs (22) had the PFAM domains PF00060.29 and PF10613.12 typical of this receptor gene family and 26 (74%) candidates mapped against an insect IR from the UniProtKB/Swiss-Prot database. Almost all sequences (32) presented 2 to 5 transmembrane domains. *Triatoma infestans* (38) had an IR repertoire similar to those of other heteropterans, such as *O. fasciatus* (37), *A. lucorum* (33), *R. prolixus* (30), and *C. lectularius* (30) (Table 1). To note, non-heteropteran insects like *N. lugens* (25), *Ac. pisum* (19), *S. furcifera* (16), and *D. citri *(6) tended to present smaller IR repertoires.

**Fig. 1:**
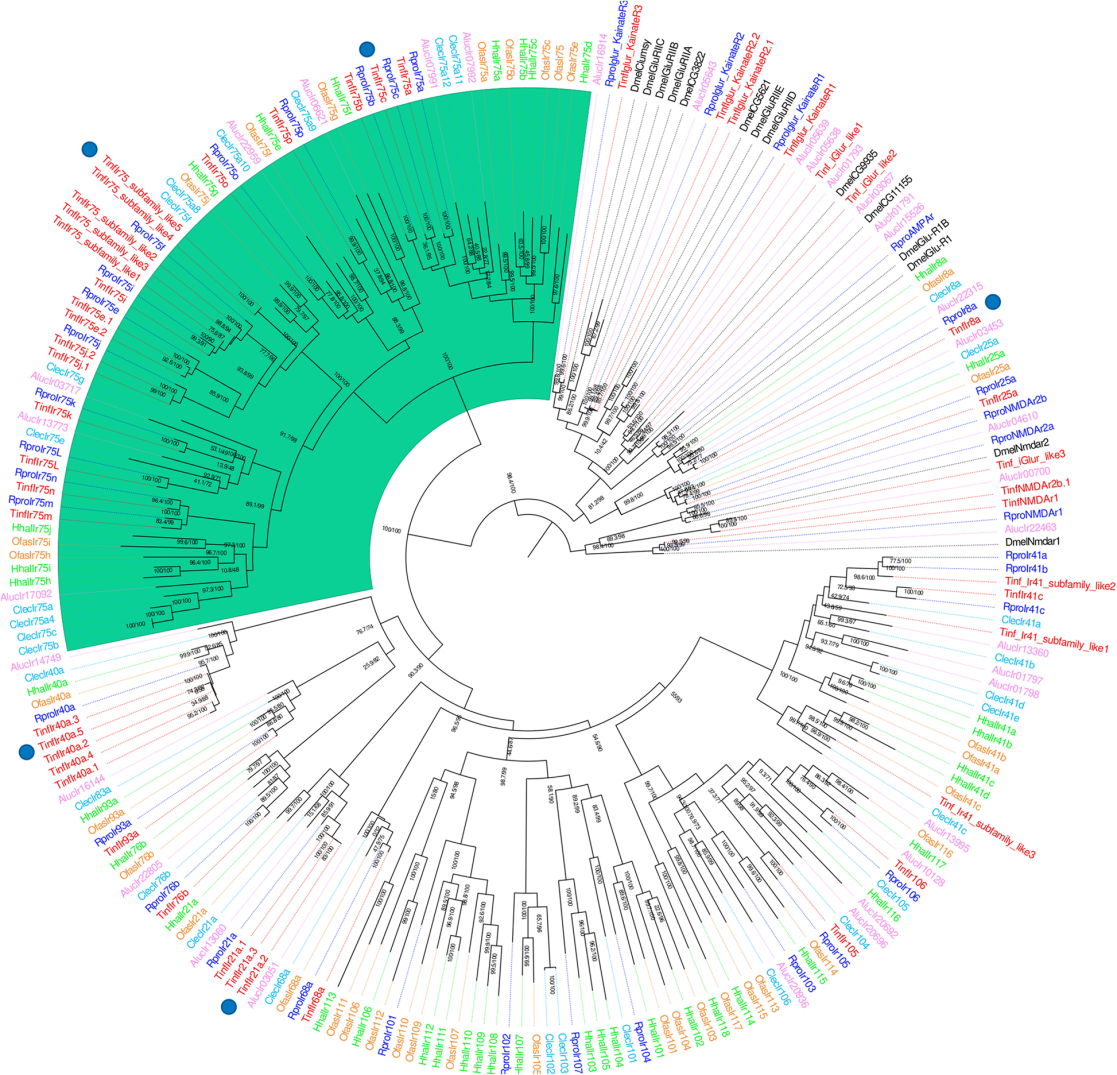
Ionotropic receptors phylogenetic tree. The maximum-likelihood tree was constructed using IQ-Tree and support values are shown for branches with both aLRT-SH and UFBoot values. Down and up-regulated transcripts after blood ingestion are marked with blue and red circles in the tree, respectively. The tree was rooted using the NMDA receptor sequences. The clade with the *Ir75* subfamily is highlighted in green. *Drosophila melanogaster* sequences were obtained from Croset et al. (2010) [51]. Aluc—*Apolygus lucorum*, Clec—*Cimex lectularius*, Dmel—*Drosophila melanogaster*, Hhal—*Halyomorpha halys*, Ofas—*Oncopeltus fasciatus*, Rpro - *Rhodnius prolixus*,  and Tinf—*Triatoma infestans*. Sequences with TRINITY IDs belong to *T. infestans*

Except for most sequences belonging to the* Ir41* subfamily and a few sequences of the *Ir75* subfamily, *T. infestans* IRs (30) were annotated based on their relationships to *R. prolixus *IRs. This was the case of the three IR co-receptors* Ir25a*, *Ir8a*, and *Ir76b*, as well as the orthologues of several antennal IRs, such as *Ir21a*, *Ir40a*, *Ir41*, *Ir68a* and *Ir93a* (Fig. 1). As observed for *R. prolixus* [41], orthologues of the other highly conserved IRs, including* Ir31a*, *Ir92a*, *Ir60a*,* Ir76a*, *Ir64a* and *Ir84a*, were not detected for *T. infestans*. Most of the IRs presented a 1:1 orthology relation between *T. infestans *and *R. prolixus*, except for *Ir40a *and* Ir21a* lineages that seem to have expanded in* T. infestans*, with 5 and 3 transcripts each (Fig. 1). Among the divergent IRs, only orthologues of *Ir105* and *Ir106* were identified for both triatomines, while the remaining were only detected for *R. prolixus*. As previously reported for *R. prolixus* (13 *Ir75* members; [41]), a large expansion of the *Ir75* subfamily was identified for *T. infestans*, with 19 paralogues and some duplicated members for *Ir75e*, *Ir75f*,* Ir75i* and *Ir75j* (Fig. 1, highlighted in green). The expansion of the *Ir75* lineage is an evolutionary process that seems to include *C. lectularius* and *O. fasciatus*, with 12 and 10 *Ir75* genes, respectively. For non-heteropteran hemipterans, this lineage seems to have contracted or had no *Ir75* orthologues, such as for *N. lugens*, *S. furcifera*, and *D. citri* (Table 1).

*TinfIr40a* (with five transcripts) and *TinfIr8a* were the most highly expressed IRs in unfed and fed antennal libraries (Additional file 7: Supplementary Figure S3). Interestingly, *TinfIr40a.5* and *TinfIr8a* were down-regulated after blood ingestion (see Results section "Effect of feeding on antennal transcript abundance").

### Gustatory receptors

The data mining of our database allowed the identification of 11 *T. infestans* GRs, with an average length of 305 amino acids, and only two of them representing complete proteins (Additional file 5: Supplementary Table S3). The number of transmembrane domains of the* T. infestans* GRs varied from 3 to 7. All GRs showed a positive hit against PF08395.15, which is the PFAM domain of the GR family. *Triatoma infestans*, with 11 GRs, presented the smallest repertoire among those from other hemipterans studied here (Table 1). Our phylogenetic analysis revealed that the GR expansion formed by *RproGr5-17* may be exclusive of *R. prolixus*, as no *T. infestans* GR was identified in this clade (Additional file 6: Supplementary Figure S2).

Almost all *T. infestans* GRs had clear *R. prolixus *orthologues, except for two sequences: *TRINITY_DN25527_c0_g2_i2.p1* that seems to be related to *Gr1* from *C. lectularius*, *H. halys*, and *O. fasciatus *and, *TRINITY_DN4868_c0_g1_i6.p1* that clustered out of the clade formed by *Gr26*, *Gr27* and *Gr28* genes from *R. prolixus* and *T. infestans* (Additional file 6: Supplementary Figure S2). Mesquita et al. (2015) [41] identified only one *R. prolixus* GR (*RproGr1*) that could have a putative sugar ligand because of sequence similarity to the *Drosophila melanogaster* fructose receptor. All other heteropterans here analyzed had an orthologue of this GR; however, we were not able to identify it in our *T. infestans *de novo assembly. As reported for *R. prolixus* [41], *T. infestans* does not seem to have orthologues of the *D. melanogaster* CO_2_ receptors.

Most *T. infestans *GRs showed low expression levels in unfed and fed bug antennae (8 out of 11 had < 1 TPM), *TinfGr27.2* being the one with highest expression (Additional file 4: Supplementary Table S2 and Additional file 7: Supplementary Figure S3). The expression of this GR increased significantly after ingestion of a blood meal (see Results section "Effect of feeding on antennal transcript abundance").

### Odorant binding proteins

A total of 41 OBPs were identified in the de novo assembled transcriptome, with an average length of 146 amino acids, and only eight of them represented incomplete transcripts (Additional file 5: Supplementary Table S3). The analysis of the OBP sequences of *T. infestans* revealed the six conserved cysteines in 28 transcripts; the characteristic conserved *alpha* helices (between 6 and 7) in 36 sequences; and the presence of the signal peptide in 28 of them. Except for one OBP candidate, the rest showed positive hits against the PFAM domain PF01395.25, typical of this gene family.

Martínez-Barnetche et al. (2018) [40] identified 34 and 41 contigs from the *Triatoma pallidipennis* and *Triatoma dimidiata* transcriptomes that could encode for OBPs. Instead, our more conservative sequence analysis based on criteria of similarity (grouping sequences presenting > 95% of identity) and length (sequences < 100 amino acids were eliminated) revealed 28 and 26 OBPs in *T. pallidipennis *and *T. dimidiata*, respectively. In the case of *O. fasciatus*, a total of 22 OBPs were identified in its predicted protein database. Out of the 14 hemipteran species compared here, only *H. halys* presented an OBP repertoire (44) slightly larger than that of *T. infestans* (Table 1). Species belonging to the Auchenorrhyncha (*N. lugens* and *S. furcifera*) and Sternorrhyncha (*Ac. pisum* and *D. citri*) suborders presented overall smaller OBP repertoires (Table 1).

**Fig. 2:**
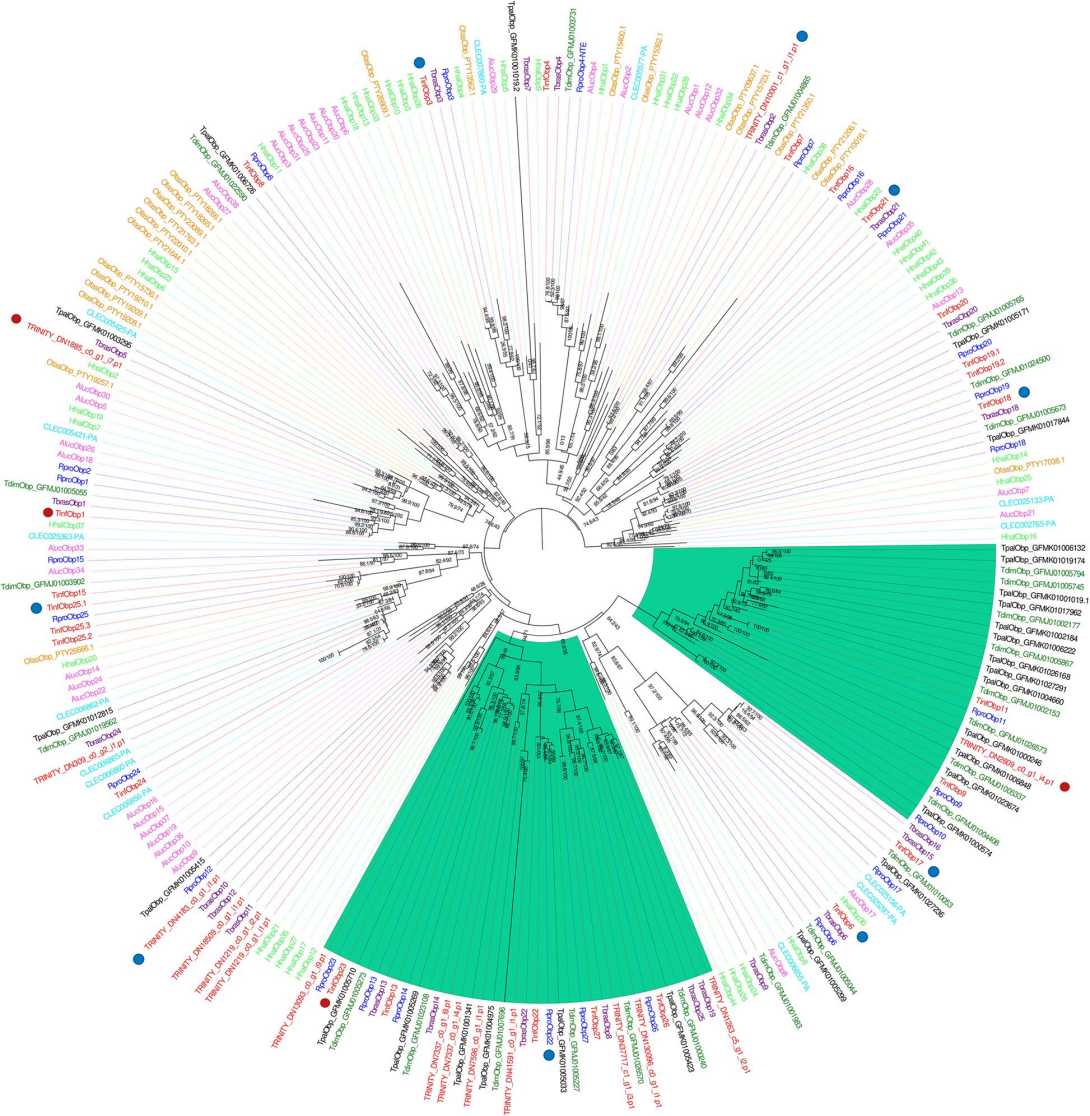
Odorant binding proteins phylogenetic tree. The maximum-likelihood tree (rooted a the midpoint) was constructed using IQ-Tree and support values are shown for branches with both aLRT-SH and UFBoot values. Down and up-regulated transcripts after blood ingestion are marked with blue and red circles in the tree, respectively. Clades that are exclusive to triatomines are highlighted in green. *Cimex lectularius* and *O. fasciatus* maintained their VectorBase and NCBI codes, respectively. In the case of *T. dimidiata* and *T. pallidipennis*, sequences were identified using the contig IDs from databases where their CDSs were obtained. Sequences from *T. brasiliensis* maintained the annotation proposed by Marchant et al*.* (2016) [38]. Aluc—*Apolygus lucorum*, Clec—*Cimex lectularius*, Hhal—*Halyomorpha halys*, Ofas—*Oncopeltus fasciatus*, Rpro - *Rhodnius prolixus*, Tbra—*Triatoma brasiliensis*, Tdim—*Triatoma dimidiata*, Tinf—*Triatoma infestans* and Tpal—*Triatoma pallidipennis*. Sequences with TRINITY IDs belong to *T. infestans*

Out of the 41 OBPs annotated for *T. infestans* based on our transcriptome, 25 presented orthology with those of *R. prolixus* (Fig. 2). The OBPs from the other triatomines kept their original IDs. Several OBPs, such as *Obp6*, *Obp14*, *Obp17*, *Obp18*, *Obp20*, *Obp22*, *Obp24*, and *Obp26*, are conserved in the five triatomine species; and some of them (*Obp6, Obp17*, *Obp6*, and *Obp24*) are even conserved within all heteropterans analyzed. Small species-specific OBP expansions (with 3 to 7 members) were observed for all species, except for *C. lectularius*. Regarding triatomines, two clades (highlighted in green) deserve attention. The first was composed mainly of OBPs of *T. dimidiata* (8) and *T. pallidipennis* (13), with few sequences of *R. prolixus* (3) and *T. infestans* (3), and none from *Triatoma brasiliensis*. The second clade has exclusive and well-conserved orthogroups from all triatomines, like those constituted by *TinfObp14*, *TinfObp22*,* TinfObp23*, *TinfObp26*, and *TinfObp27* and their corresponding orthologues.

Two separated groups could be visualized when OBP expression was depicted for the 12 antennal libraries (Additional file 7: Supplementary Figure S3). The first group included 20 OBPs highly expressed in bug antennae (all of them with an expression > 28 TPM in all libraries), clearly higher than those of ORs, GRs and IRs. A total of 7 OBPs in this group had expression levels above 1,000 TPM, for example, *TinfObp6*, *TinfObp17*, and *TinfObp18* (Additional file 4: Supplementary Table S2 and Additional file 7: Supplementary Figure S3). The second group included OBPs with low antennal expression.

### Chemosensory proteins

**Fig. 3:**
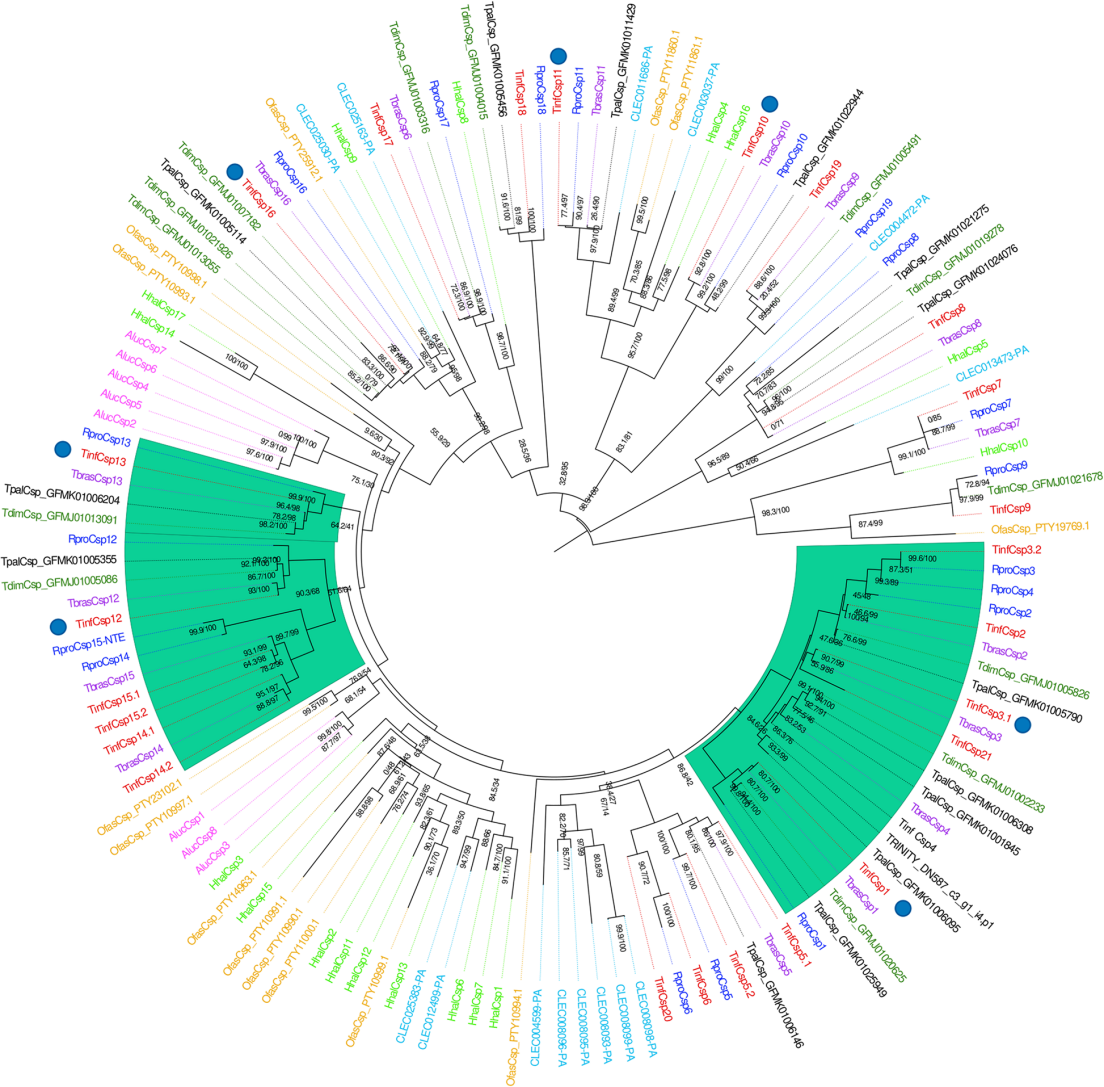
Chemosensory proteins phylogenetic tree. The maximum-likelihood tree (rooted at the midpoint) was constructed using IQ-Tree and support values are shown for branches with both aLRT-SH and UFBoot values. Down and up-regulated transcripts after blood ingestion are marked with blue and red circles in the tree, respectively. Clades that are exclusive to triatomines are highlighted in green. *Cimex lectularius* and *O. fasciatus* maintained their VectorBase and NCBI codes, respectively. In the case of *T. dimidiata* and *T. pallidipennis*, sequences were identified using the contig IDs from databases where their CDS were obtained. Sequences from *T. brasiliensis* maintained the annotation proposed by Marchant et al. (2016) [38]. Aluc—*Apolygus lucorum*, Clec—*Cimex lectularius,* Hhal—*Halyomorpha halys*, Ofas—*Oncopeltus fasciatus*, Rpro - *Rhodnius prolixus*, Tbra—*Triatoma brasiliensis*, Tdim—*Triatoma dimidiata*, Tinf—*Triatoma infestans* and Tpal—*Triatoma pallidipennis*. Sequences with TRINITY IDs belong to *T. infestans*

Nineteen CSP transcripts were found through data mining the assembled transcriptome, most of them complete (only three are partial transcripts), having four cysteines and between 6 to 7 *alpha* helices in their sequences (Additional file 5: Supplementary Table S3). All CSP sequences had the PFAM domain PF03392.16, while the signal peptide was absent in only two of them. The *TinfCsp4*, *TinfCsp6*, *TinfCsp9*, *TinfCsp18*, *TinfCsp19*, and *TinfCsp22* sequences described by Traverso et al. (2022) [28] for *T. infestans* were not reconstructed in our antennal transcriptome. On the other hand, three new CSPs were identified in our assembly (Additional file 5: Supplementary Table S3). Martínez-Barnetche et al. (2018) [40] reported 23 and 24 contigs that could encode CSPs based on *T. pallidipennis* and *T. dimidiata* transcriptomes, respectively. As with OBPs, our more conservative analysis identified only 14 and 13 CSPs, respectively. A total of 14 CSP sequences were obtained from the predicted protein database of *O. fasciatus*. Interestingly, the CSP repertoire was largest in *T. infestans* (25 sequences) among the hemipterans, considering data herein and those of Traverso et al. (2022) [28] (Table 1). This was also observed for OBPs. Representatives of the Auchenorrhyncha and Sternorrhyncha suborders had the smallest repertoires (Table 1). Following the pattern seen for OBPs, several CSP clades were conserved and contained orthologues among heteropteran species, like those including *TinfCsp8*, *TinfCsp9*, *TinfCsp11*, or *TinfCsp16*. On the other hand, others represented specific expansions, for example, the clade of *C. lectularius* formed by *CLEC008095*, *CLEC008096*, and 5 additional CSPs from *C. lectularius* (Fig. 3). Several CSP lineages (highlighted in green in the tree) seem to be exclusive of triatomines and were highly conserved among all species included here, such as *TinfCsp4*, *TinfCsp12*, or *TinfCsp13*.

Only 4 CSPs presented < 1 TPM in the conditions tested here (Additional file 4: Supplementary Table S2). As for OBPs, several CSPs were highly expressed, presenting expression values > 1000 TPM (Additional file 4: Supplementary Table S2 and Additional file 7: Supplementary Figure S3). Besides, the expression of several CSPs was also significantly affected by ingestion of a blood meal, e.g., *TinfCsp3.1* and *TinfCsp11* (see Results section "Effect of feeding on antennal transcript abundance").

### *Takeouts*

The *takeout* (TO) repertoire of *T. infestans* was composed of 36 candidates (Additional file 5: Supplementary Table S3), all of them having the PF06585.14 domain that is characteristic of this family. Only 5 TOs presented partial sequences and all of them presented very similar lengths (247 amino acids on average). Seventeen new TO genes were identified in the *R. prolixus* genome (annotated from *RproTo17* to *RproTo32*), resulting in a total of 32 (Additional file 5: Supplementary Table S3).

*Triatoma infestans* and *R. prolixus* seem to have expanded TO repertoires (36 and 32 members, respectively) compared to other triatomine species (Table 1). *Apolygus lucorum* (56) and *H. halys* (46) have the largest TO repertoires (Table 1) due to their specific expansions (Additional file 6: Supplementary Figure S2). Almost all *R. prolixus* TOs had a clear *T. infestans* orthologue. Furthermore, orthogroups were conserved in the case of the triatomines (*To2*,* To6*, *To11,* and *To29* lineages) presenting representatives for all five species included here.

Most TOs were expressed in unfed and fed antennal bug samples, with more than half having expression values > 20 TPM, while only 5 had expression levels < 1 TPM (Additional file 4: Supplementary Table S2 and Additional file 7: Supplementary Figure S3). A group of 10 TOs showed high expression in bug antennae with expression values > 500 TPM. A highlight should be made for *TinfTo29 *(28,888 TPM in unfed antennae) whose expression was significantly decreased after blood ingestion (see Results section "Effect of feeding on antennal transcript abundance").

### Transient receptor potential channels

We identified 17 TRP sequences in the antennal transcriptome database (Additional file 5: Supplementary Table S3), 9 of them were complete and all of them had clear *R. prolixus* orthologues (Additional file 6: Supplementary Figure S2). Compared to *R. prolixus*, *T. infestans* had two transcripts for *inactive* (*Iav*), *Trp-gamma*, *TrpA1*, *TrpA5*, and *TrpM*. The TRP repertoire of *T. infestans* (17) is larger than those of *R. prolixus* (14), *Ac. pisum* (13), and *D. melanogaster* (13), and only *A. lucorum* (21) and *H. halys* (19) seemed to present more TRP genes in their genomes (Table 1). Each TRP gene was located in separated and well-defined branches of the phylogenetic tree and members from the different TRP subfamilies were grouped as previously described [52]. As expected considering the conservation of these receptors across insect evolution [52, 53], orthologues of almost all TRP genes were identified for the heteropterans studied here. However, orthologues of *Trp* and *TrpC* genes were not found in triatomines, while heteropterans do not seem to have the TRP channel named *Pyrexia* (*Pyr*).

Most of the TRPs presented low expression (< 1.5 TPM) in the 12 antennal libraries, except for *TinfPain*, *TinfTrpML*, *TinfTrpM1*, *TinfTrpA5.2* and *Tinfwtrw*, the transcripts of which were detected in greater abundance (Additional file 4: Supplementary Table S2 and Additional file 7: Supplementary Figure S3).

### *Pickpocket* receptors

A total of 13 PPK sequences were found in our transcriptome database (Additional file 5: Supplementary Table S3). All of them presented the typical PFAM domain PF00858.2 of the amiloride-sensitive sodium channel family. Seven of these sequences were complete and most of them presented transmembrane domains (ranging between 1 to 4). All *T. infestans* PPKs presented clear *R. prolixus *orthologues, including those of *ppk23*, *ppk9* (with two transcripts), and *ppk28* (Additional file 6: Supplementary Figure S2). *Halyomorpha halys* had the larger PPK repertoire (16) of all heteropterans included here (Table 1).

These receptors mostly showed low expression (< 1 TPM) levels in bug antennae, except for *TinfPPK subfamily (subf) V like-1 *which had an expression value of 34.15 and 42.21 TPM in unfed and fed buh antennae, respectively, followed by *TinfPPK subfII like-1* and *TinfPPK subfV like-2* (Additional file 4: Supplementary Table S2 and Additional file 7: Supplementary Figure S3).

### Other sensory genes

Three SNMPs, 1 CheA, 5 CheBs, and 3 ammonium transporters (including the orthologues of* Rh50*, *AmT1*, and *AmT3*) were identified in our transcriptomic database (Additional file 5: Supplementary Table S3). Regarding SNMPs, *T. infestans* had 2 Snmp1 and 1 Snmp2 transcripts, while *R. prolixus* had 2 *Snmp1* and 2 *Snmp2* genes (Additional file 6: Supplementary Figure S2). All heteropterans, except for *O. fasciatus* which seemed to lack SNMP orthologues, presented members of the *Snmp1* orthogroup. *Halyomorpha halys* was the only non-triatomine species presenting an *Snmp2* gene. The expression of SNMP genes in our transcriptome was higher than that detected for other sensory receptor families (Additional file 4: Supplementary Table S2). *TinfRh50* and *TinfAmT1* presented gene expression > 1 TPM in the 12 libraries (Additional file 4: Supplementary Table S2 and Additional file 7: Supplementary Figure S3).

According to our phylogenetic analysis, *T. infestans* presented 6 CHE sequences. Particularly, one of them was included within the CheA clade and the remaining are related to *RproCheB* (Additional file 6: Supplementary Figure S2). CheA sequences are absent from *R. prolixus* and *O. fasciatus* protein databases, while we did not find any CHE for *C. lectularius*. *TinfCheB1* was the most highly expressed member of this gene family (59 and 42 TPM in unfed and fed bug antennae, Additional file 4: Supplementary Table S2 and Additional file 7: Supplementary Figure S3) and the abundance of its transcripts was significantly reduced after the ingestion of a blood meal.

### Effect of feeding on antennal transcript abundance

An average of 41.7 M reads *per* library was obtained after the trimming and cleaning step (Additional file 1: Supplementary Table S1). Out of them, 21.1 M reads on average *per* library mapped against the non-redundant CDS database. The resulting count matrix (38,291 transcripts) was filtered so that transcripts with low expression and/or high variability were removed. The resulting matrix (with 9,963 transcripts) was used as input for differential expression analysis (Additional file 8: Supplementary Table S4). A Principal Component Analysis showed the consistency of our dataset, as each treatment’s replicates (6) clustered together and apart from the other condition (Additional file 9: Supplementary Figure S4).

To analyze whether a blood meal ingestion induced changes in gene expression profiles of *T. infestans* antennae we defined significance and fold change thresholds to restrict our analysis to the most relevant alterations (s-values < 0.05 and an absolute fold-change threshold > 1.5). Blood ingestion induced a great modification of the gene expression profile of *T. infestans* antennae, with 2,122 transcripts (more than 20% of all included transcripts) significantly changing their abundance (Additional file 10: Supplementary Table S5). From this set of differentially expressed transcripts, 1,186 were down-regulated (518 of them with a fold-change > 100%) and 936 were up-regulated (456 of them with a fold-change > 100%). The nucleotide sequence, protein sequence, GO-terms [54], KEGG-terms [55, 56], top results of BLASTp searches against SwissProt/UniProt and PFAM databases, and the last version of the annotated proteins of the *R. prolixus* genome for each differentially expressed transcript were included in the Additional file 11: Supplementary Table S6.

The results of the GO-enrichment analysis confirmed that the ingestion of blood caused a great impact on the antennal physiology. A total of 235 enriched GO-terms from the molecular function, biological process, and cellular component categories were found (Additional file 12: Supplementary Table S7). The terms “behavioral response to starvation” and “feeding behavior” were among those enriched according to the analysis.

**Fig. 4:**
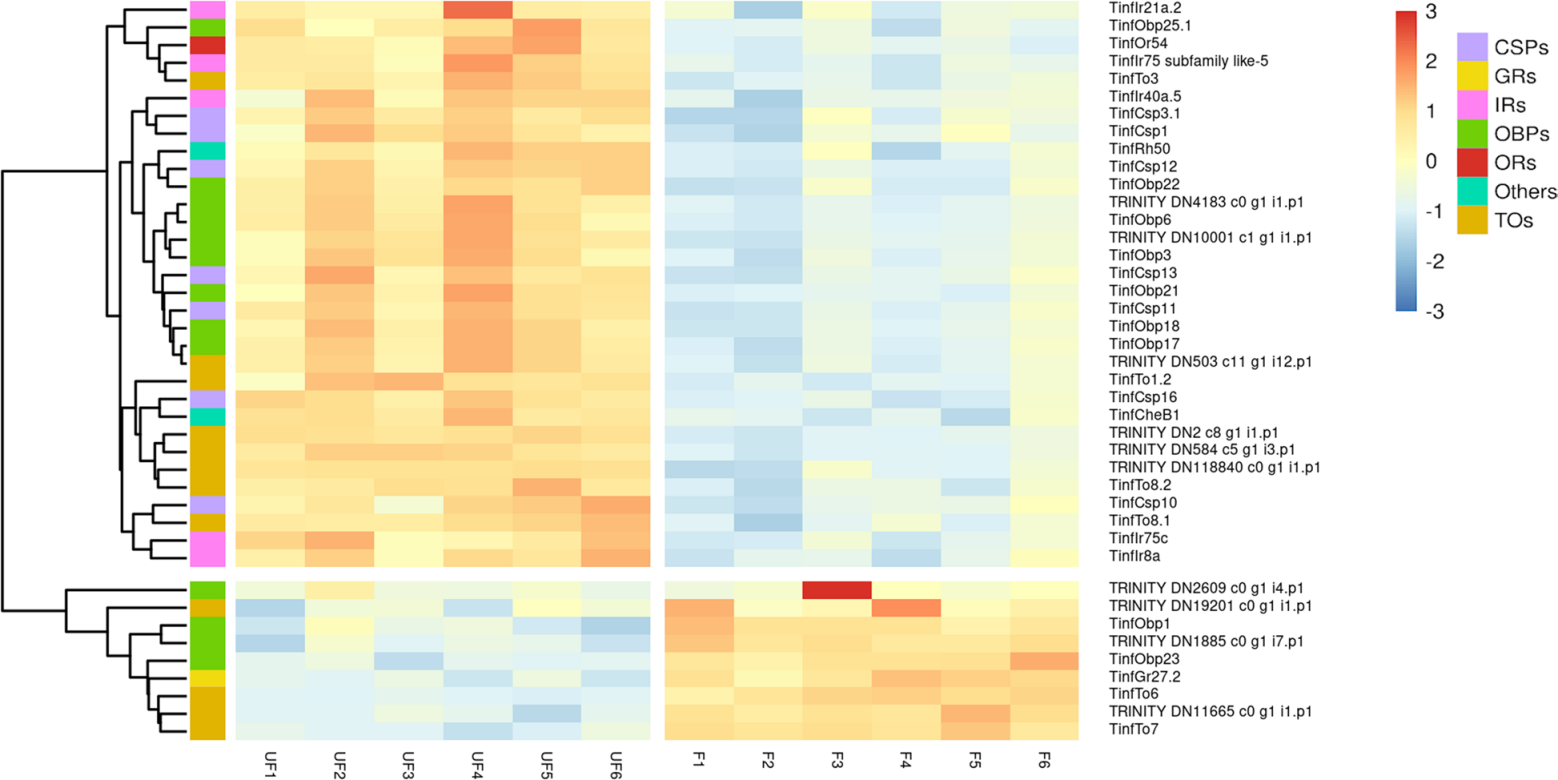
Effect of feeding on the antennal transcription of sensory genes. The heatmap was created using transformed count data (generated with *rlog* function in DESeq2 and included in Additional file 13: Supplementary Table S8) as input of the pheatmap R package that calculated a Z-score for each gene and plotted it by means of a color scale, where blue/red represent the lowest/highest expression. UF: unfed bug samples (control) and F: fed bug samples

The ingestion of a blood meal by *T. infestans* significantly affected the antennal transcript abundance of 41 genes directly related to insect sensory processes (Fig. 4 and Additional file 13: Supplementary Table S8). One OR (*TinfOr54*), 5 IRs (*TinfIr8a*, *TinfIr75_subfamily_like5*, *TinfIr21a.2*, *TinfIr75c*, and *TinfIr40a.5*), 9 OBPs, 7 CSPs, 8 TOs, the ammonium transporter *TinfRh50*, and *TinfCheB1* decreased their transcript abundance in the antennae after blood ingestion. The number of up-regulated transcripts post-blood meal was much lower: 4 OBPs, 4 TOs, and 1 GR (*TinfGr27.2*).

The phylogenetic analysis of the odorant carriers (OBPs and CSPs) and TOs whose expression was affected after blood ingestion showed that several of these genes are conserved across all included triatomines (Fig. 2, Fig. 3 and Additional file 6: Supplementary Figure S2) like *TinfObp6*, *TinfObp17*, *TinfObp18*, and *TinfObp22* (Fig. 2), or *TinfCsp1*, *TinfCsp12* and *TinfCsp13* (Fig. 3) or *TinfTo16* and *TinfTo29* (Additional file 6: Supplementary Figure S2). Indeed, genes like *TinfObp6*, *TinfObp17*, and *TinfTo16* were found to be conserved for heteropterans, as their orthologues were also identified for *A. lucorum*, *H. halys*, *C. lectularius*, and *O. fasciatus*.

## Discussion

Our work establishes the basis of molecular research on the sensory processes of *T. infestans*, as we describe the sensory gene repertoire of this relevant Chagas disease vector species. As part of this characterization, we report a set of sensory genes whose antennal expression is modulated by the ingestion of a blood meal. We suggest that they mediate triatomine host-seeking and propose functional studies to determine their specific roles in the detection of multimodal host cues. Therefore, these genes represent potential targets for bug behavioral manipulation for more sustainable control of Chagas disease in the future.

Additionally, our study presents novel facts about the evolutionary history of diverse molecular components underlying heteropteran sensory processes, allowing us to identify sensory solutions developed by this diverse lineage of hemimetabolan insects. We report heteropteran-specific gene clades, as well as triatomine-specific ones, for several families of sensory-related genes. In the future, it would be necessary to include more sequences from other heteropteran and non-heteropteran insects to corroborate the existence of these heteropteran-specific lineages.

### Feeding impacts the antennal expression of sensory-related genes

The responsiveness of *R. prolixus* to host-associated cues, such as CO_2_ and heat, and the motivation to feed decreases after blood ingestion [57]. A reduction in the antennal abundance of Orco and the IR-coreceptor transcripts triggered by feeding, probably underlying these behavioral changes, was reported in *R. prolixus* [58]. However, studies about specific sensory receptors and/or odorant carriers involved in host-seeking behavior in triatomines are very limited so far [59, 60]. Antennal RNA sequencing (RNA-Seq) has proven excellent to identify gene candidates mediating host cue detection by detecting transcriptional changes triggered by blood ingestion [17, 20–22]. Therefore, our study also aimed at uncovering such genes in *T. infestans*.

The effect of blood ingestion on OR and GR expression was extremely slight, as only two of these chemoreceptors had their antennal expression significantly altered after feeding (Fig. 4 and Additional file 13: Supplementary Table S8). Coincidentally, the ingestion of a blood meal had subtle effects on the antennal expression of ORs in *Aedes aegypti* [21] and *Anopheles gambiae* [17], while in the case *Culex quinquefasciatus* and *Culex pipiens* a blood meal seemed to impose larger effects [20, 22]. In the case of *TinfOr54*, a significant down-regulation was observed (Fig. 4), transforming it into a promising candidate to mediate the detection of a host-related odor cue. On the other hand, the already high antennal expression of *TinfGr27* increased significantly after blood ingestion (Fig. 4). Interestingly, previous RNA-Seq experiments revealed that the *R. prolixus* orthologue of this receptor also had a high antennal expression level under diverse conditions [18, 42], suggesting a relevant role in triatomine sensory physiology.

The effect of a blood meal was more pronounced on the antennal expression of IRs, as the transcript abundance of five of these receptors decreased significantly after bug feeding (Fig. 4 and Additional file 13: Supplementary Table S8). The members of the *Ir75 *clade have been implicated in the detection of aliphatic amines [61] and short-chain fatty acids in *D. melanogaster* [61] and *An. gambiae* [62], and these volatile compounds have also been identified in triatomine host odors [5, 63–67]⁠. These facts and the down-regulation observed for *TinfIr75c* and *TinfIr75-like5* after blood ingestion point to a potential role of these receptors in mediating triatomine host-seeking behavior. Similar expression changes of several *Ir75s* have been observed 24 h after blood-feeding in the antennae of  *Cx. quinquefasciatus* [20], suggesting a functional connection. The antennal expression of *TinfIr8a* was also significantly reduced after blood ingestion, coinciding with previous results observed in *R. prolixus* [58]. Indeed, in *Ae. aegypti* the* Ir8a* pathway is required to detect lactic acid and the other acidic components of human sweat [68]. Lactic acid has been described as a potent synergist for the orientation of *T. infestans* to CO_2_-laden airstreams [51]. Therefore, it is possible that there is a conserved role in host recognition for the Ir8a-dependent olfactory subsystem in these highly divergent insect blood feeders.

Triatomines and other vectors of human pathogens rely on diverse sensory modalities besides olfaction to find their hosts. The thermal sense is fundamental for triatomine host-seeking [23, 69]; however, its molecular bases have been poorly studied [59]. As observed in mosquitoes [20], the abundance of antennal transcripts for *TinfIr21a* decreased after blood feeding (Fig. 4 and Additional file 13: Supplementary Table S8). Interestingly, this IR drives heat-seeking and heat-stimulated blood feeding in *An*. *gambiae* [26] and is required for cool avoidance in *D. melanogaster* [70]. These functional data together with the high degree of conservation of *Ir21a* across insects [47] turn it into a candidate to mediate the detection of host-emitted thermal cues by kissing bugs. A fundamental role in host-seeking has also been attributed to triatomine [25, 71] and mosquito [72] hygrosensation. Studies in *D. melanogaster* have shown that several IRs (*Ir93a*, *Ir40a*, *Ir25a* and *Ir68a*) are required for sensing changes in environmental humidity [73–75]. These facts and the significantly reduced expression of *TinfIr40a* (Fig. 4 and Additional file 13: Supplementary Table S8) after blood feeding suggest that this IR may act as a triatomine hygroreceptor mediating host-seeking.

Unlike with ORs, IRs and GRs, the effect of feeding over the expression of the odorant carrier proteins was more prominent, significantly altering the expression of 13 OBPs and 7 CSPs (Fig. 4 and Additional file 13: Supplementary Table S8). In a previous RNA-Seq experiment targeting *R. prolixus* antennae, a significantly altered expression of odorant carrier proteins through post-ecdysis age was also more prevalent than on sensory receptors [18]. A similar pattern has been observed in other antennal transcriptomes analyzing nutritional and developmental effects in mosquitoes [17, 20–22]. This strongly suggests that changes in odorant carrier expression levels could be relevant to regulate sensory function. In the cited study, several *R. prolixus* OBPs and CSPs had their antennal expression significantly increased after ecdysis and clearly correlated with the increased responsiveness to host cues seen during the first week after molting [18]. Interestingly, *T. infestans* orthologues of some of these odorant carriers (*TinfCsp11*, *TinfCsp13*, *TinfObp18*, *TinfObp21* and *TinfObp22*) were significantly down-regulated after bug feeding (Fig. 4). Therefore, interfering with the function of representatives of this set of odorant carriers, and especially the highly conserved *Csp11*, *Obp18* and* Obp22* (Fig. 2 and Fig. 3), represents a promising strategy to affect triatomine host-seeking abilities.

Some TO genes have been reported to act as carriers of juvenile hormone (JH), therefore relating to larval molting and reproduction [76, 77]. The ability of proteins of the TO family to carry JH has also been pointed to relate to food intake and foraging behavior, among other functions [77–79]. The antennal expression of 12 *T. infestans* TOs was modulated by feeding (Fig. 4 and Additional file 13: Supplementary Table S8), similarly to what was observed in the antennae of *Spodoptera littoralis* [80]. This TO subgroup, and especially the TOs whose expression decreased after feeding (Fig. 4), may prove relevant to regulate triatomine foraging behavior and/or feeding. The *TinfTo29* gene would be the first candidate to test this hypothesis considering its high transcript abundance (Additional file 4: Supplementary Table S2), the pronounced decrease in expression it presented after feeding (Additional file 13: Supplementary Table S8), and its conservation among the five triatomine species studied here (Additional file 6: Supplementary Figure S2).

Ammonia (NH_3_) is attractive for *R. prolixus* and *T. infestans* [66, 67], and it is present in triatomine host excreta [81], human breath and sweat [82]. Interestingly, *R*. *prolixus* larvae display reduced electrophysiological responses to this compound after ingesting a blood meal [83]. The detection of NH_3_ in *D. melanogaster* and *An. gambiae* is mediated by antennal ORNs that express the *AmT* and *Rh50* genes [84–86]. Considering the high level of conservation of this transporter among diverse organisms [84, 86, 87] and that the antennal expression of *TinfRh50* decreased after blood feeding (Fig. 4 and Additional file 13: Supplementary Table S8), it is possible to suggest that it mediates NH_3_a detection in the host-seeking context of kissing bugs.

Blood ingestion induced a huge impact on the antennal expression profile of *T. infestans*, as more than 1,000 genes had their abundance altered at least two-fold after feeding (Additional file 10: Supplementary Table S5). Similar results were observed when the antennal transcriptomes of unfed and fed mosquitoes were compared [17, 20–22]. In this work, we have focused on the analysis of sensory related-genes; however, feeding could affect other gene families potentially involved in sensory physiology, such as neuropeptides, neuropeptide and neurotransmitter receptors, odorant degrading enzymes, or nuclear receptors [17, 19, 21]. Therefore, the expression profiles of these targets deserve to be studied in our dataset in the future. In addition, our study focused on the transcriptional effects that had been triggered in the antennae of the fifth instar larvae four days post blood ingestion. Antennal RNA samples generated at different times after feeding would probably show more complex expression profiles. Finally, it is important to consider that neural [16] and molecular changes taking place in the central nervous system [19] and other sensory structures e.g. rostrum and tarsi [19, 88], could be acting in parallel in the modulation of triatomine host-seeking behavior.

### The sensory repertoire of *T. infestans*

Besides transcriptome quantification, RNA-Seq provides transcript sequences that allow phylogenetic and comparative analyses in organisms lacking genomic annotated sequences, as is the case of *T. infestans*. Since the publication of the *R. prolixus* genome [41], new hemipteran genomes and transcriptomes have been sequenced, allowing a better understanding of the evolution of sensory gene families within this largest hemimetabolan insect order. In this sense, our phylogenetic analyses identified new triatomine-specific lineages, as well as conserved orthogroups for sensory gene families within Heteroptera.

The sensory gene repertoires of *T. infestans* and *R. prolixus* revealed to be very similar, at least in terms of gene number (Table 1). This might seem puzzling, considering their largely different habitats e.g. palm trees *vs*. rocky mounds, and geographical distribution. Nevertheless, genomic information for *T. infestans* is still necessary to confirm the size and complete identity of the sensory gene families reported here.

The phylogenetic trees obtained for the different sensory gene families showed similar evolutionary patterns (Fig. 1–3 and Additional file 6: Supplementary Figure S2). This included expanded clades that were species (the large GR clades from *H. halys* or *O. fasciatus*), or subfamily-specific (such as some OBP and CSP orthogroups observed for triatomines), or that included sequences from very different heteropteran families, like the *TinfOr104* orthogroup. This pattern is consistent with the gene birth-and-death model that drives the evolution of these rapidly evolving genes [89].

In the case of the ORs, we identified six subfamily-exclusive gene clades that expanded through the evolution of triatomines (highlighted in green in Additional file 6: Supplementary Figure S2), suggesting that they mediate bug-specific behaviors, such as pheromone detection or host-seeking. Interestingly, the largest triatomine clade (*RproOr58-87* and its orthologues in *T. infestans*) is composed of young ORs, probably originating from recent gene duplication events. Indeed, most of these ORs belong to a single tandem array in a few scaffolds of the *R. prolixus* genome [41], reinforcing their recent evolutionary origin. On the other hand, several old OR lineages of single genes previously identified in *R. prolixus* (*RproOr1*, *RproOr2*, *RproOr101-105*, *RproOr107*, and *RproOr112*) [41] were also found in *T. infestans* and other heteropterans (Additional file 6: Supplementary Figure S2). Functional information for some of these old OR lineages has been generated in *C. lectularius* [90, 91]. For instance, *ClecOr5* (related to *TinfOr103* and *RproOr103*) responds to nonanal and octanal; and *ClecOr9* (related to *TinfOr105* and *RproOr105*) detects decanal. These compounds have been identified in triatomine hosts, such as humans, sheep and chickens, and elicit behavioral and electrophysiological responses in *T. infestans* and *R. prolixus* [5], suggesting that aldehydes may be the cognate ligands of these triatomine ORs.

Orthologues of the three IR co-receptors (*Ir8a*, *Ir25a*, and *Ir76b*) and the well-conserved antennal IRs were identified for *T. infestans* and all other heteropterans (Fig. 1). Except for *Ir41* and several* Ir75s*, *T. infestans* and *R. prolixus* showed a clear 1:1 orthologue relation for most of the IRs. Orthologues of some antennal IRs, including *Ir31a*, *Ir92a*, *Ir60a*, *Ir76a*, *Ir64a* and *Ir84*a [47] were absent in all heteropteran databases studied here, a phylogenetic feature yet unknown. The great expansion observed for the *Ir75* lineage in triatomines and *C. lectularius* represents almost half of all IRs found in these insects (Table 1). This is not the case for other insects presenting expanded *Ir75 *lineages like the mosquitoes *Ae. aegypti* (12 *Ir75s* out of 135 IRs) [92] and *Cx. quinquefasciatus* (13 *Ir75s* out of 61) [20]; or *Blattodea* representatives like *Blattella germanica* (26 *Ir75s* out 455) [93] and *Zootermopsis nevadensis* (17 *Ir75s* out of 141) [94]. This particular pattern and the fact that two *Ir75s* are down-regulated after blood ingestion (Fig. 4) turns these receptors into interesting candidates to perform functional genetic studies and to apply cheminformatic pipelines to evaluate their role in triatomine sensory processes.

A reduced number of GR transcripts (11) was identified in our *T. infestans* antennal transcriptome (Table 1). A high divergence at the sequence level that may have reduced the efficiency of our search strategy, and the low antennal expression (8 out of 11 had < 1 TPM) potentially explains the low number of GRs identified for *T. infestans*. A future annotation of the genomic sequence of *T. infestans* should allow us to evaluate whether this reduction reported for its GR numbers is real. The phylogenetic analysis shows that the youngest lineage among the *R. prolixus* GRs (*RproGr5-17*) [41] seems to be exclusive of this species, but this might also be biased by the limited access to triatomine GR data mentioned above.

*Triatoma infestans* presents the largest CSP repertoire (25) among the hemipterans studied here. A similar case was seen for OBPs, as the repertoire of this species (41) was only below that of *H. halys* (44) (Table 1). Heteropteran OBP numbers were clearly higher than those of Auchenorrhyncha and Sternorrhyncha, while the number of CSPs seems to be more homogeneous (Table 1). Nevertheless, a great variation in the numbers of these binding proteins has been observed across insect orders, without any correlation to their ecological and nutritional preferences or taxonomic relationships [95]⁠. Regarding TRPs,* Trp* and *TrpC* genes from the TRPC subfamily were found to be absent in triatomines. The *Trp *gene mediates phototransduction (reviewed by [96]) and mutant *D. melanogaster* flies for this and other TRPC subfamily members (*TrpL* and *Trp-gamma*) show reduced sensitivity to CO_2_ [97].

In spite of using an antennal transcriptome with a high sequencing depth (> 500 M cleaned and trimmed reads, Additional file 1: Supplementary Table S1), sensory receptors or odorant transporters of *T. infestans* with low expression i.e. gustatory receptors; or expressed in other physiological conditions may not be identified in our transcriptome. Several transcripts were not completely reconstructed and genomic information will be necessary to complete and validate their sequences. In addition, whether transcripts whose sequences were very similar are isoforms, allelic variants, or they come from different genes will only be determined after the genomic sequence and gene annotation of *T. infestans* becomes available. Besides, sequencing more triatomine and hemipteran genomes would help to confirm the particular evolutionary features described in this work for sensory-related gene families within the order Hemiptera, and especially within Chagas disease vectors.

### New sensory genes were identified in heteropterans

We have annotated several sensory protein families (CheAs, CheBs, CSPs, PPKs, SNMPs and TOs) of diverse heteropterans (Table 1 and Additional file 6: Supplementary Figure S2). This will allow functional genetic studies to be performed in those species. Our phylogenetic analysis confirms that heteropterans lack PPKs from subfamilies IV (formed by *ppk2*2 and *ppk24* genes, among others) and VII (composed by *ppk17*) and that instead the highly conserved *ppk9*, *ppk23*, and *ppk28* prevail within this insect suborder [98]. This is the first report to study in detail the TO gene family of Heteroptera, identifying sequences of 7 species and improving the annotation of *R. prolixus* and *T. brasiliensis* TO repertoires. Interestingly, heteropterans had larger TO repertoires compared to other insects [99]. The *Pyr* gene, a TRP related to thermal physiology, was absent from the databases of all heteropterans studied here. This thermosensor has been shown to detect high temperatures (close to 40 °C) helping to protect insects from harmful warm conditions [100, 101]. Therefore, assessing the molecular mechanism through which heteropterans deal with such noxious stimuli seems appealing. On the other hand, no SNMP or CHE members were identified in the *O. fasciatus* and *C. lectularius* predicted protein databases, respectively. SNMPs have been described in many insect species and seem to be involved in pheromone and odor detection (revised by [102]). The negative BLAST searches against the *O. fasciatus* genome using the SNMP sequences of *Nysius ericae* [103] allowed us to conclude that the former species lacks these proteins.

## Conclusions

Feeding significantly impacted the gene expression profiles of 20% of the genes expressed in *T. infestans* antennae. Furthermore, most of these genes were downregulated after ingesting a blood meal. Interestingly, a relevant proportion of these genes can be connected to sensory function. In conclusion, several gene candidates to mediate the detection of host-associated cues by triatomines have been identified, creating opportunities to develop behavioral manipulation strategies based on them in the future. Nevertheless, to follow on the observed expression changes, functional genetic studies i.e. RNA interference coupled to behavioral experiments or heterologous expression systems would still be necessary to characterize their roles. Furthermore, it will be fundamental to evaluate whether the transcriptional changes observed in the antennae imply modifications at the level of sensory neuron responsiveness.

## Material and methods

### Insects

Fifth instar larvae of *T. infestans* were obtained from colony X32 of the Centro de Referencia de Vectores (CeReVe) of Argentina and reared under controlled conditions (26 ± 1 °C temperature and a 12 h:12 h light/dark cycle). This colony was established at CeReVe with field-captured insects from Santiago del Estero province (Argentina) five years before our samples were obtained. For our experiment testing the effect of blood feeding, two groups of fifth instar larvae were sorted immediately after ecdysis. Half of them were kept unfed until using them for sample preparation, while the remaining bugs were fed ad libitum on pigeons at day 30 post-ecdysis. Antennal samples from both groups of insects (unfed and fed) were obtained on day 34 after ecdysis. Each condition was replicated 6 times using 20 antennae *per* sample. Antennae were homogenized manually using sterilized pestles and total RNA was extracted from the homogenate using the TRIzol® Reagent (Life Technologies, Carlsbad, CA, USA). The extracted RNA was resuspended in 25 µL of DEPC-treated water (Life Technologies). RNA integrity and quality were assessed by means of a 1% agarose electrophoresis gel and in an Agilent 2100 Bioanalyzer (Agilent Technologies, Santa Clara, CA, USA).

Pigeons used in this work to feed the bugs were housed, cared, fed, and handled following the resolution 1047/2005 (Consejo Nacional de Investigaciones Científicas y Técnicas, CONICET, Argentina) regarding the national reference ethical framework for biomedical research with laboratory, farm, and nature collected animals (Marco Ético de Referencia para las Investigaciones Biomédicas en Animales de Laboratorio, de Granja y Obtenidos de la Naturaleza), which is in accordance with the standard procedures of the Office for Laboratory Animal Welfare, Department of Health and Human Services, NIH and the recommendations defined by the 2010/63/EU Directive of the European Parliament, related to animals used for scientific purposes and National Law 14,346 on Animal Welfare. The final protocol was evaluated by a committee in the Regional Center of Genomic Studies (CREG) at the National University of La Plata (Argentina), which confirmed the accordance with the ethical frameworks. Biosecurity considerations are in agreement with CONICET resolution 1619/2008, which is in accordance with the WHO Biosecurity Handbook (ISBN 92 4 354 6503).

### Sequencing and read processing

Library construction and high-throughput sequencing services were hired at Macrogen (Seul, South Korea). A total of 12 cDNA libraries (six *per* experimental condition) were generated using the TruSeq Stranded mRNA LT Sample Prep Kit from Illumina (San Diego, CA, USA). The libraries were sequenced using an Illumina NovaSeq 6000 equipment with paired-end reads of 150 base-pair (bp) length. The raw data set is available at SRA Bioproject number PRJNA882044. The FASTQC tool (available at www.bioinformatics.babraham.ac.uk/projects/fastqc) was used to evaluate read quality and the presence of Illumina sequencing adapters in the raw reads. To remove low-quality bases from 5’ and 3’ ends and sequencing adapters, we used Trimmomatic (v0.39) in the paired-end mode with the following settings: TRAILING: 5, LEADING: 5, ILLUMINACLIP: TruSeq3-PE-2.fa:2:30:10 and SLIDING-WINDOW 4:15. Reads shorter than 50 bp were eliminated.

### De novo assembly

The trimmed and cleaned reads from the 12 libraries were used to generate a de novo transcriptome assembly by means of the Trinity software (v.2.10.0), with the option* –SS_lib_type RF *used for this type of stranded libraries. Afterward, the *TrinityStats.pl script* was used to obtain the basic statistics of the assembly. A non-redundant CDS database of the assembled transcripts was generated following the pipeline used by Traverso et al. (2022) [28] and Tanaka et al. (2022) [104]. First, open reading frames (ORFs) with a length of at least 100 amino acids were predicted using the *TransDecoder.LongOrfs* script from the TransDecoder software v5.5.0 (http://transdecoder.github.io). Second, BLASTp (v2.9.0 +) and HMMscan (v3.3) searches were carried out on the predicted ORFs using the complete UniProtKB/Swiss-Prot and PFAM-A databases as queries. Third, the output of these searches was used in the *TransDecoder.Predict* script to generate the predicted CDSs. Finally, those CDSs with > 95% of sequence identity were clustered and filtered using the *cd-hit-est* script of CD-HIT software (v.4.8.1). The resulting non-redundant CDS database was used to perform the differential expression analysis, and the protein sequences obtained from its translation were used to identify the sensory gene repertoire of *T. infestans*. The completeness of the non-redundant translated CDS database was evaluated by means of BUSCO (v4.1.4) in protein mode against the hemiptera_odb10 lineage data set.

### Identification of sensory genes

#### Identification and sequence analysis of sensory gene candidates of *T. infestans*

BLASTp searches on the non-redundant translated CDS database were performed using the following queries: 1) The *R. prolixus* sensory gene sequences previously described by Mesquita et al. (2015) [41] and Latorre-Estivalis. (2017, 2020) [18, 43]; 2) The OR, IR and GR sequences from *C. lectularius* [37], *A. lucorum* [38, 105], *O. fasciatus* [32] and *H. halys* [33]; 3) The PFAM seed sequences in fasta format (unaligned) for each family, except for CHEs; and 4) The TRP, CHE, and PPK sequences of *D. melanogaster* and *R. prolixus*. BLAST results were filtered by sequence identity > 30% and e-value < 1 × 10^–7^ and only those sequences presenting an alignment length higher than 100 amino acids for OBP, CSP, and TO genes and 200 amino acids for the rest of the gene families were included. In addition, HMMscan searches using HMM PFAM profiles for each target family were performed on the non-redundant translated CDS database to identify additional candidates.

Sequences of sensory gene candidates were analyzed by means of 1) BLASTp searches against UniProtKB/Swiss-Prot; 2) HMMscan searches using the Pfam-A database as query; 3) Presence of transmembrane domains using the TMHMM software (v2.0), and 4) Presence of a signal peptide using SignaIP (v.5.0). The output of these searches was integrated using Trinotate v3.2.1 (https://trinotate.github.io), with an e-value 1e^−5^ to extract the positive hits in the BLAST searches, and using the default parameter “domain noise cut-off” as a criterion to select PFAM results. Using *R. prolixus* as a reference, sequences of interest were manually examined and edited if clear assembly errors were observed (e.g. a sequence was split into two assembled transcripts) or the initial methionine was not correctly predicted. These manually curated sequences were used for the phylogenetic analyses described below. Transcripts with coverage to the *R. prolixus* reference sequences > 90% were considered complete. Clustal Omega was used to align the predicted *T. infestans* CSPs and OBPs to a *Mamestra brassicae* CSP (Uniprot ID Q9NG96) and the *D. melanogaster* lush-PA sequences, respectively, in order to assess the presence of conserved residues of both protein families. PSIPRED v4.0 was used to predict CSP and OBP secondary structures. Transcripts *Per* kilobase per Million reads (TPM) values were calculated and used in the gplot package (v.3.1.1) to create heatmaps for the different sensory gene families.

#### Identification of sensory gene candidates in other heteropterans

We obtained the predicted protein databases from *O. fasciatus* (official gene set v1.2, GCA_000696205.1_Ofas_1.0_protein from i5k), *A. lucorum* (official gene set v1, GCA_009739505.2_ASM973950v2_protein from NCBI), *H. halys* (official gene set v2.0, GCF_000696795.2_Hhal_2.0_protein from NCBI) and *C. lectularius* (official gene set ClecH1.3 at VectorBase). Besides, the assembled transcriptomes of *T. brasiliensis* ([39], available at European Nucleotide Archive—ENA with ID HADI01000000.1), *T. pallidipennis* (available at NCBI with ID GFMK00000000.1) and *T. dimidiata* (available at NCBI with ID GFMJ00000000.1) from Martínez-Barnetche et al. (2018) [40] were also obtained.

*Rhodnius prolixus* and *D. melanogaster* TO, CheA, CheB, TRP, PPK, SNMP sequences and their corresponding PFAM seeds (in unaligned fasta format) were used as queries in BLASTp searches against the predicted protein databases of the four non-triatomine species with their genomes available. In the case of *O. fasciatus*, OBP and CSP sequences from the other heteropterans studied here [33–35, 39, 41] were used as queries to identify candidates from both gene families as they were not identified in the genome project. Results of BLASTp searches were processed using the same identity and length criteria previously mentioned for *T. infestans*. Besides, HMMscan searches using HMM PFAM profiles for each target family were performed on the predicted protein databases to identify additional candidates. Only the longest isoforms from the candidate sequences identified in *C. lectularius*, *H. halys* and *O. fasciatus* databases were included in the phylogenetic analyses. As no information about isoforms was available for the *A. lucorum* genome, all sequences meeting the criteria were used for the species.

Contigs containing OBP and CSP sequences were extracted from the *T. pallidipennis* and *T. dimidiata* transcriptomes (IDs previously described by Martínez-Barnetche et al., 2018 [40], and their predicted CDSs were extracted using the *TransDecoder.LongOrfs* and *TransDecoder.Predict* scripts from the TransDecoder software v5.5.0 and filtered using CD-HIT as described in the previous section. Only protein sequences longer than 100 amino acids were maintained. *Rhodnius prolixus* TO sequences [43] and the PFAM seed PF01395 (unaligned fasta format) were used in tBLASTn searches to identify *T. pallidipennis* and *T. dimidiata* candidates in their corresponding transcriptomes. Subsequently, TO CDSs were extracted using the same strategy as for OBPs and CSPs. Considering the type of samples (whole-body) used to generate the *T. pallidipennis* and *T. dimidiata* transcriptomes [40], sensory gene searches were restricted to highly expressed gene families such as OBPs, CSPs and TOs. In the case of *T. brasiliensis*, OBP, CSP and TO nucleotide sequences previously reported [39] were extracted from the corresponding database, translated to amino acids and filtering using CD-HIT software. It is important to note that contigs for *TbrasObp23* and *TbrasOpb17* reported in that study were not found in their published database, CDS extracted from *Contig17773* had less than 100 amino acids, *mira_454_rep_c72681* (partial sequence of *Contig11061*) and *comp171165_c2* (identical to *Supercontig_454_5739*) were not considered for our analyses.

Finally, the seed (small and curated set of sequences from representative members of the family) and HMM of the PFAM domain PF01395 were used in BLASTp and HMMscan searches, respectively, to identify new TO candidates in the *R. prolixus* genome predicted protein database (official gene annotation RproC3.5, available at VectorBase). To validate the absence of SNMP genes in the genome of *O. fasciatus*, SNMP sequences of *N. ericae* [103], which is another *Lygaeidae*, were used for BLASTp and tBLASTn searches.

#### Phylogenetic analyses

The protein sequences belonging to each sensory gene family were aligned using MAFFT with the G-INS-i strategy and the following settings: unaligned level = 0.1; offset value = 0.12; maxiterate = 1000 and the option "leave gappy regions". After trimming (using trimAl v1.2 by default except for the gap threshold = 0.3), the alignments were used to build phylogenetic trees on IQ-tree (v1.6.12). The branch support was estimated using both the approximate Likelihood Ratio Test based on the Shimodaira-Hasegawa (aLRT-SH) and the ultrafast bootstrap (UFBoot) approximation [106, 107]. ModelFinder was used to establish the best-fit amino acid substitution models and chosen according to the Bayesian Information Criterion. These models were JTT + F + R9 for ORs, JTT + F + R10 for GRs, WAG + F + R9 for IRs, LG + R5 for CSPs, VT + R5 for OBPs, LG + F + R7 for TOs, WAG + F + R7 for PPKs, VT + F + R6 for TRPs, LG + I + G4 for SNMPs and WAG + F + I + G4 for CHEs. The phylogenetic trees were visualized and edited with FigTree. Sensory gene candidates were annotated based on their relationship with those of *R. prolixus*. *Triatoma infestans* candidates without a clear relationship with *R. prolixus* genes retained their TRINITY codes. 

### Transcript quantification and differential expression analysis

The Salmon software (v1.4.0), using the *align_and_estimate_abundance.pl* Trinity script with the* –SS_lib_type RF* parameter, was used to map the trimmed reads from each library to the non-redundant CDS database. The matrix of counts *per* transcript for each library was obtained using the *abundance_estimates_to_matrix.pl *Trinity script. Following this procedure, transcripts showing large variation and/or low expression were eliminated from the matrix using HTSFilter v.1.28 in RStudio. The filtered matrix was used as input for DESeq2 (v.1.28.0) to perform the differential expression analysis in RStudio using the apeglm estimation from the *lfcShrink* function. This function is based on the “Approximate Posterior Estimation for Generalized Linear Model'' (apeglm) method that uses an adaptive Bayesian shrinkage estimator to generate more accurate fold-change values [108]. Differentially expressed genes (DEGs) were established using an s-value < 0.05 as the significance threshold and a fold-change threshold > 1.5 (equal to an expression change of 50%). The sequence of those genes that fitted these criteria were subsequently submitted to BLASTp searches against UniProtKB/Swiss-Prot and the predicted proteins from *R. prolixus* (release 51, downloaded from VectorBase). In addition, HMMscan searches were carried out using the Pfam-A database. The results of these analyses were integrated using Trinotate v3.2.1 (https://trinotate.github.io) using the same criteria described in the section “Identification and sequence analysis of sensory gene candidates of *T. infestans*” of the "Identification of sensory genes" section.

The enrichment analysis was carried out with ermineR R package [109] using the Gene Score Resampling (GSR) method and the s-values for each transcript to produce a score rank. This analysis was complementary to that of the differential expression, and all transcripts of the non-redundant translated CDS database (38,291 transcripts) were considered along with their corresponding s-values. GO-terms with corrected p-value lower than 0.05 were identified as enriched. For details on the strategy used see https://erminej.msl.ubc.ca/help/tutorials/running-an-analysis-resampling/. It is worth mentioning that performing a GO-enrichment analysis can have certain limitations in non-model insects like triatomines and in de novo assemblies, where the background used to identify the enriched terms is limited to the genes expressed in the tissue and conditions tested.

## Supplementary Information

**Additional file 1: Supplementary Table S1.** Sequencing output and results of the cleaning and mapping steps for the 12 samples. 

**Additional file 2: Supplementary Figure S1. **Graphical summary of the BUSCO assessment results of the non-redundant translated CDS database against Hemiptera.

**Additional file 3: Supplementary Data file S1. **Fasta protein sequences of sensory-related genes of *T. infestans* and other insects that were used in the phylogenetic analyses.

**Additional file 4: Supplementary Table S2. **Expression, represented as Log_10_ (TPM+1), of the sensory-related genes in the 12 libraries. TPM: Transcripts *Per* kilobase *per* Million reads.

**Additional file 5: Supplementary Table S3. **Description of transcripts, annotation, manual curation, the top results of BLASTp searches against SwissProt/UniProt, the output of *hmmscan* searches by HMMER (Pfam column), presence of signal peptide, number of predicted transmembrane domains, their associated GO and KEGG terms, nucleotide and protein sequences for ORs (A), IRs (B), GRs (C), OBPs (D), CSPs (E), TOs (F), TRPs (G), PPKs (H) SNMPs (I), and CHEs (J).

**Additional file 6: Supplementary Figure S2. **Phylogenetic trees of sensory-related gene families analyzed. The maximum-likelihood trees were constructed using IQ-Tree and support values are shown for branches with both aLRT-SH and UFBoot values. Down and up-regulated transcripts after blood ingestion are marked with blue and red circles in the trees, respectively. The OR phylogenetic tree (A) was rooted using *Orco* and OR expansions that are exclusive to triatomines are highlighted in green. The GR phylogenetic tree (B) was rooted at the midpoint. The TO (C) phylogenetic tree was rooted at the midpoint. *Cimex lectularius *and *O. fasciatus* maintained their VectorBase and NCBI codes, respectively. In the case of *T. dimidiata *and *T. pallidipennis*, sequences were identified using the contig IDs from databases where their CDSs were obtained. *Takeout* sequences kept their assembly codes (if we were able to find them in their database). The TRP phylogenetic tree (D) was rooted at the midpoint. *Apolygus lucorum*, *C. lectularius *and *O. fasciatus* TRPs maintained their NCBI and VectorBase codes, while *D. melanogaster* sequences were obtained from Matsuara et al*.* (2009) [52]. The PPK phylogenetic tree (E) was rooted using *Dmelppk17*. *Apolygus lucorum*, *C. lectularius *and *O. fasciatus* PPKs maintained their NCBI and VectorBase codes, while *D. melanogaster* sequences were obtained from Zelle et al*.* (2013) [110]. The CHE phylogenetic tree (F) was rooted at the midpoint. *Apolygus lucorum *and *O. fasciatus* CheAs maintained their NCBI codes and* D. melanogaster* CheA and CheB sequences were obtained from Torres-Oliva et al. (2016) [111]. The SNMP phylogenetic tree (G) was rooted at the midpoint. Aluc - *Apolygus lucorum*, Clec - *Cimex lectularius*, Dmel - *Drosophila melanogaster*, Hhal - *Halyomorpha halys, *Ofas - *Oncopeltus fasciatus*, Rpro - *Rhodnius* *prolixus*, Tbra - *Triatoma brasiliensis*, Tdim - *Triatoma dimidiata*, Tinf - *Triatoma infestans* and Tpal - *Triatoma pallidipennis*. Sequences with TRINITY IDs belong to *T. infestans*.

**Additional file 7: Supplementary Figure S3. **Transcript abundance of the *T. infestans *sensory-related gene families. OR (A), IR (B), GR (C), OBP (D), CSP (E), TO (F), TRP (G), PPK (H) SNMP (I), ammonium transporter (J) and CHE (K) heatmaps were created using Log_10_ (Transcripts *Per* kilobase *per* Million reads +1) as input of the gplot R package. Transcript abundance was represented in a color scale where white/red represents the lowest/highest expression. A dendrogram was plotted using hierarchical clustering of genes based on euclidean distances and a complete linkage method for clustering. UF: unfed bug samples (control) and F: fed bug samples.

**Additional file 8: Supplementary Table S4.** Raw count matrix generated by Salmon software after mapping trimmed and cleaned reads against the non-redundant CDS database. UF: unfed bug samples (control) and F: fed bug samples.

**Additional file 9: Supplementary Figure S4. **Principal Component Analysis graph.

**Additional file 10: Supplementary Table S5. **Results of the differential expression analysis performed in DESeq2. Differentially expressed genes with an absolute fold-change > 2 (LogFc > 1) are presented using bold font, while genes showing an absolute fold-change > 1.5 (LogFc > 0.58) are presented in blue.

**Additional file 11: Supplementary Table S6.** Description of differentially expressed transcripts. The top results of BLASTp searches against SwissProt/UniProt, the output of *hmmscan* searches by HMMER (Pfam column), and the last official gene annotation from the *R. prolixus* genome (available at VectorBase), their associated GO and KEGG terms, and nucleotide and protein sequences are included.

**Additional file 12: Supplementary Table S7. **Statistics of GO-enrichment analysis. Enriched GO-terms are presented in bold font (corrected p-value < 0.05).

**Additional file 13: Supplementary Table S8. **Statistics of differentially expressed transcripts with putative sensory functions. The corresponding normalized (generated using  the *rlog *function from DESeq2) and raw counts are presented for all libraries. Genes with an absolute fold-change > 2 (LogFc > 1) are presented in bold font. UF: unfed bug samples (control) and F: fed bug samples.

## Data Availability

The datasets generated and/or analysed during the current study are available in the repository at Sequence Read Archive (SRA) from NCBI with the Bioproject number PRJNA882044. Available at https://www.ncbi.nlm.nih.gov/bioproject/PRJNA882044.

## Abbreviations

ORs: Odorant receptors
IRs: Ionotropic receptors
GRs: Gustatory receptors
OBPs: Odorant binding proteins
CSPs: Chemosensory proteins
ODEs: Odorant-degrading enzymes
SNMPs: Sensory neuron membrane proteins
OSNs: Olfactory sensory neurons
TRPs: Transient receptor potential channels
PPKs: *Pickpockets*
AmTs: Ammonium transporters
CDS: Coding sequence (CDS)
BUSCOs: Benchmarking Universal Single-Copy Orthologues
TPM: Transcripts *P**er* kilobase per Million reads
iGluRs: Ionotropic glutamate receptors
TOs: *Takeouts*
CHEs: Chemosensory proteins
GO: Gene Ontology
KEGG: Kyoto Encyclopedia of Genes and Genomes
RNA-Seq: RNA sequencing
HMM: Hidden Markov Model
aLRT: Approximate Likelihood Ratio Test
SH: Shimodaira-Hasegawa-like

## References

[1] WHO. Chagas disease in Latin America: an epidemiological update based on 2010 estimates. Wkly Epidemiol Rec. 2015(90):33–44. 25671846

[2] Gürtler RE, Pilar Fernández M del, Cardinal MV. Eco-epidemiology of vector-borne transmission of *Trypanosoma cruzi* in domestic habitats. In: Triatominae - The biology of Chagas disease vectors. Springer; 2021. p. 447–89.

[3] Gürtler RE, Cecere MC. Chagas disease vector control. In: Triatominae - The biology of Chagas disease vectors. Springer; 2021. p. 491–535.

[4] Hansson BS, Stensmyr MC (2011). Evolution of insect olfaction. Neuron.

[5] Guerenstein PG, Guerin PM. Olfactory and behavioural responses of the blood-sucking bug *Triatoma infestans* to odours of vertebrate hosts. J Exp Biol. 2001;204:585–97. 10.1242/jeb.204.3.585 11171309

[6] Takken W (1991). The role of olfaction in host-seeking of mosquitoes: a review. Int J Trop Insect Sci.

[7] Manrique G, Lorenzo M. The sexual behaviour of Chagas’ disease vectors: chemical signals mediating communication between male and female Triatomine bugs. 2012;Psyche (Stuttg):1–8.

[8] Allison JD, Carde RT. Pheromone communication in moths: evolution, behavior, and application. Univ of California Press; 2016.

[9] Khallaf MA, Cui R, Weißflog J, Erdogmus M, Svatoš A, Dweck HK, et al. Large-scale characterization of sex pheromone communication systems in *Drosophila*. Nat Commun. 2021;12:1–14. 10.1038/s41467-021-24395-z PMC8260797 34230464

[10] Basu S, Clark RE, Fu Z, Lee BW, Crowder DW (2021). Insect alarm pheromones in response to predators: Ecological trade-offs and molecular mechanisms. Insect Biochem Mol Biol.

[11] Verheggen FJ, Haubruge E, Mescher MC (2010). Alarm pheromones—chemical signaling in response to danger. Vitam Horm.

[12] Seiedy M, Saboori A, Zahedi-Golpayegani A. Olfactory response of *Phytoseiulus persimilis* (Acari: Phytoseiidae) to untreated and *Beauveria bassiana*-treated *Tetranychus urticae* (Acari: Tetranychidae). Exp Appl Acarol. 2013;60:219–27. 10.1007/s10493-012-9652-8 23271063

[13] Mburu D, Ochola L, Maniania N, Njagi P, Gitonga L, Ndung’u M, et al. Relationship between virulence and repellency of entomopathogenic isolates of *Metarhizium anisopliae* and *Beauveria bassiana* to the termite *Macrotermes michaelseni*. J Insect Physiol. 2009;55:774–80. 10.1016/j.jinsphys.2009.04.015 19442668

[14] Carey AF, Carlson JR (2011). Insect olfaction from model systems to disease control. Proc Natl Acad Sci U S A.

[15] Leal WS. Odorant reception in insects: roles of receptors, binding proteins, and degrading enzymes. Annu Rev Entomol. 2013;58:373–91. 10.1146/annurev-ento-120811-153635 23020622

[16] Gadenne C, Barrozo RB, Anton S. Plasticity in insect olfaction: to smell or not to smell? Annu Rev Entomol. 2016;61:317–33. 10.1146/annurev-ento-010715-023523 26982441

[17] Rinker DC, Pitts RJ, Zhou X, Suh E, Rokas A, Zwiebel LJ. Blood meal-induced changes to antennal transcriptome profiles reveal shifts in odor sensitivities in *Anopheles gambiae*. Proc Natl Acad Sci U S A. 2013;110:8260–5. 10.1073/pnas.1302562110 PMC3657813 23630291

[18] Latorre-Estivalis JM, Große-Wilde E, da Rocha FG, Hansson BS, Lorenzo MG. Changes in antennal gene expression underlying sensory system maturation in* Rhodnius prolixus*. Insect Biochem Mol Biol. 2022;140:103704. 10.1016/j.ibmb.2021.103704 34942331

[19] Matthews BJ, McBride CS, DeGennaro M, Despo O, Vosshall LB. The neurotranscriptome of the* Aedes aegypti *mosquito. BMC Genomics. 2016;17:32. 10.1186/s12864-015-2239-0 PMC4704297 26738925

[20] Taparia T, Ignell R, Hill SR (2017). Blood meal induced regulation of the chemosensory gene repertoire in the southern house mosquito. BMC Genomics.

[21] Hill SR, Taparia T, Ignell R. Regulation of the antennal transcriptome of the dengue vector, *Aedes aegypti*, during the first gonotrophic cycle. BMC Genomics. 2021;22(1):71. 10.1186/s12864-020-07336-w PMC7821643 33478394

[22] Gu Z, Gao H, Yang Q, Ni M, Li M, Xing D, et al. Screening of olfactory genes related to blood-feeding behaviors in *Culex pipiens quinquefasciatus* and *Culex pipiens molestus* by transcriptome analysis. PLoS Negl Trop Dis. 2022;16:e0010204. 10.1371/journal.pntd.0010204 PMC8853563 35130307

[23] Flores GB, Lazzari CR. The role of the antennae in *Triatoma infestans*: Orientation towards thermal sources. J Insect Physiol. 1996.

[24] Corfas RA, Vosshall LB (2015). The cation channel TRPA1 tunes mosquito thermotaxis to host temperatures. eLife.

[25] Barrozo RB, Manrique G, Lazzari CR. The role of water vapour in the orientation behaviour of the blood-sucking bug *Triatoma infestans* (Hemiptera, Reduviidae). J Insect Physiol. 2003;49:315–21. 10.1016/s0022-1910(03)00005-2 12769985

[26] Greppi C, Laursen WJ, Budelli G, Chang EC, Daniels AM, van Giesen L (2020). Mosquito heat seeking is driven by an ancestral cooling receptor. Science.

[27] Rinker DC, Pitts RJ, Zwiebel LJ (2016). Disease vectors in the era of next generation sequencing. Genome Biol.

[28] Traverso L, Latorre Estivalis JM, da Rocha FG, Fronza G, Lobbia P, Mougabure Cueto G, et al. Transcriptomic modulation in response to an intoxication with deltamethrin in a population of *Triatoma infestans* with low resistance to pyrethroids. PLoS Negl Trop Dis. 2022;16:e0010060. 10.1371/journal.pntd.0010060 PMC9275713 35767570

[29] Calderón-Fernández GM, Moriconi DE, Dulbecco AB, Juárez MP. Transcriptome analysis of the *Triatoma infestans* (Hemiptera: Reduviidae) integument. J Med Entomol. 2017;54:1531–42. 10.1093/jme/tjx151 29029205

[30] Buarque DS, Braz GR, Martins RM, Tanaka-Azevedo AM, Gomes CM, Oliveira FA, et al. Differential expression profiles in the midgut of *Triatoma infestans* infected with *Trypanosoma cruzi*. PLoS ONE. 2013;8:e61203. 10.1371/journal.pone.0061203 PMC3642171 23658688

[31] Assumpção TC, Francischetti IM, Andersen JF, Schwarz A, Santana JM, Ribeiro JM. An insight into the sialome of the blood-sucking bug *Triatoma infestans*, a vector of Chagas’ disease. Insect Biochem Mol Biol. 2008;38:213–32. 10.1016/j.ibmb.2007.11.001 PMC2262853 18207082

[32] Panfilio KA, Vargas Jentzsch IM, Benoit JB, Erezyilmaz D, Suzuki Y, Colella S (2019). Molecular evolutionary trends and feeding ecology diversification in the Hemiptera, anchored by the milkweed bug genome. Genome Biol.

[33] Sparks ME, Bansal R, Benoit JB, Blackburn MB, Chao H, Chen M, et al. Brown marmorated stink bug, *Halyomorpha halys* (Stål), genome: Putative underpinnings of polyphagy, insecticide resistance potential and biology of a top worldwide pest. BMC Genomics. 2020;21:1–26. 10.1186/s12864-020-6510-7 PMC7071726 32171258

[34] Sun D, Huang Y, Qin Z, Zhan H, Zhang J, Liu Y, et al. Identification of candidate olfactory genes in the antennal transcriptome of the stink bug *Halyomorpha halys*. Front Physiol. 2020;11:876. 10.3389/fphys.2020.00876 PMC7394822 32792985

[35] Sparks ME, Shelby KS, Kuhar D, Gundersen-Rindal DE. Transcriptome of the invasive brown marmorated stink bug, *Halyomorpha halys* (Stål) (heteroptera: Pentatomidae). PLoS ONE. 2014;9(11):e111646. 10.1371/journal.pone.0111646 PMC4227672 25386688

[36] Wu ZZ, Qu MQ, Pu XH, Cui Y, Xiao WY, Zhao HX, et al. Transcriptome sequencing of *Tessaratoma papillosa* antennae to identify and analyze expression patterns of putative olfaction genes. Sci Rep. 2017;7:1–11. 10.1038/s41598-017-03306-7 PMC5465196 28596537

[37] Benoit JB, Adelman ZN, Reinhardt K, Dolan A, Poelchau M, Jennings EC (2016). Unique features of a global human ectoparasite identified through sequencing of the bed bug genome. Nat Commun.

[38] Liu Y, Liu H, Wang H, Huang T, Liu B, Yang B, et al. *Apolygus lucorum* genome provides insights into omnivorousness and mesophyll feeding. Mol Ecol Resour. 2020;21(1):287–300. 10.1111/1755-0998.13253 32939994

[39] Marchant A, Mougel F, Jacquin-joly E, Costa J, Almeida E, Harry M. Under-expression of chemosensory genes in domiciliary bugs of the Chagas disease vector *Triatoma brasiliensis*. PLoS Negl Trop Dis. 2016;10(10):e0005067. 10.1371/journal.pntd.0005067 PMC5085048 27792774

[40] Martínez-Barnetche J, Lavore A, Beliera M, Téllez-Sosa J, Zumaya-Estrada FA, Palacio V (2018). Adaptations in energy metabolism and gene family expansions revealed by comparative transcriptomics of three Chagas disease triatomine vectors. BMC Genomics.

[41] Mesquita RD, Vionette-Amaral RJ, Lowenberger C, Rivera-Pomar R, Monteiro FA, Minx P, et al. Genome of *Rhodnius prolixus*, an insect vector of Chagas disease, reveals unique adaptations to hematophagy and parasite infection. Proc Natl Acad Sci U S A. 2015. 10.1073/pnas.1506226112 PMC4672799 26627243

[42] Latorre-Estivalis JM, Robertson HM, Walden KKO, Ruiz J, Gonçalves LO, Guarneri AA, et al. The molecular sensory machinery of a Chagas disease vector: Expression changes through imaginal moult and sexually dimorphic features. Sci Rep. 2017. 10.1038/srep40049 PMC5216343 28059141

[43] Latorre-Estivalis JM, Sterkel M, Ons S, Lorenzo MG. Transcriptomics supports local sensory regulation in the antenna of the kissing-bug *Rhodnius prolixus*. BMC Genomics. 2020. 10.1186/s12864-020-6514-3 PMC6993403 32000664

[44] Zhou S-S, Sun Z, Ma W, Chen W, Wang M-Q. De novo analysis of the *Nilaparvata lugens* (Stål) antenna transcriptome and expression patterns of olfactory genes. Comp Biochem Physiol Part D Genomics Proteomics. 2014;9:31–9. 10.1016/j.cbd.2013.12.002 24440828

[45] He P, Engsontia P, Chen G-L, Yin Q, Wang J, Lu X, et al. Molecular characterization and evolution of a chemosensory receptor gene family in three notorious rice planthoppers, *Nilaparvata lugens*, *Sogatella furcifera* and *Laodelphax striatellus*, based on genome and transcriptome analyses. Pest Manag Sci. 2018;74:2156–67. 10.1002/ps.4912 29542232

[46] Wang L, Tang N, Gao X, Chang Z, Zhang L, Zhou G, et al. Genome sequence of a rice pest, the white-backed planthopper (*Sogatella furcifera*). GigaScience. 2017;6:1–9. 10.1093/gigascience/giw004 PMC5437944 28369349

[47] Croset V, Rytz R, Cummins SF, Budd A, Brawand D, Kaessmann H (2010). Ancient protostome origin of chemosensory ionotropic glutamate receptors and the evolution of insect taste and olfaction. PLoS Genet..

[48] Richards S, Gibbs RA, Gerardo NM, Moran N, Nakabachi A, Stern D, et al. Genome sequence of the pea aphid *Acyrthosiphon pisum*. PLoS Biol. 2010;8(2):e1000313. 10.1371/journal.pbio.1000313 PMC2826372 20186266

[49] Zhou JJ, Vieira FG, He XL, Smadja C, Liu R, Rozas J, et al. Genome annotation and comparative analyses of the odorant-binding proteins and chemosensory proteins in the pea aphid *Acyrthosiphon pisum*. Insect Mol Biol. 2010;19(SUPPL. 2):113–22. 10.1111/j.1365-2583.2009.00919.x 20482644

[50] Wu Z, Zhang H, Bin S, Chen L, Han Q, Lin J. Antennal and abdominal transcriptomes reveal chemosensory genes in the Asian Citrus Psyllid *Diaphorina citri. *PLoS ONE. 2016;11:1–23. 10.1371/journal.pone.0159372 PMC4956155 27441376

[51] Barrozo RB, Lazzari CR. Orientation behaviour of the blood-sucking bug *Triatoma infestans* to short-chain fatty acids: synergistic effect of L-lactic acid and carbon dioxide. Chem Senses. 2004;29:833–41. 10.1093/chemse/bjh249 15574819

[52] Matsuura H, Sokabe T, Kohno K, Tominaga M, Kadowaki T (2009). Evolutionary conservation and changes in insect TRP channels. BMC Evol Biol.

[53] Peng G, Shi X, Kadowaki T (2015). Evolution of TRP channels inferred by their classification in diverse animal species. Mol Phylogenet Evol.

[54] Consortium GO (2004). The Gene Ontology (GO) database and informatics resource. Nucleic Acids Res..

[55] Kanehisa M, Goto S. KEGG: Kyoto encyclopedia of genes and genomes. Nucleic Acids Res. 2000;28:27–30. 10.1093/nar/28.1.27 PMC102409 10592173

[56] Kanehisa M, Furumichi M, Sato Y, Kawashima M, Ishiguro-Watanabe M. KEGG for taxonomy-based analysis of pathways and genomes. Nucleic Acids Res. 2022:1–6 10.1093/nar/gkac963 PMC9825424 36300620

[57] Bodin A, Vinauger C, Lazzari CR. Behavioural and physiological state dependency of host seeking in the blood-sucking insect *Rhodnius prolixus*. J Exp Biol. 2009;212(Pt 15):2386–93. 10.1242/jeb.030668 19617431

[58] Latorre-Estivalis JM, Omondi BA, DeSouza O, Oliveira IHR, Ignell R, Lorenzo MG. Molecular basis of peripheral olfactory plasticity in *Rhodnius prolixus*, a Chagas disease vector. Front Ecol Evol. 2015.

[59] Zermoglio PF, Latorre-Estivalis JM, Crespo JE, Lorenzo MG, Lazzari CR. Thermosensation and the TRPV channel in *Rhodnius prolixus*. J Insect Physiol. 2015;81:145–56. 10.1016/j.jinsphys.2015.07.014 26225467

[60] Pontes G, Latorre-Estivalis JM, Gutiérrez ML, Cano A, de Astrada MB, Lorenzo MG (2022). Molecular and functional basis of high-salt avoidance in a blood-sucking insect. iScience.

[61] Prieto-Godino LL, Rytz R, Cruchet S, Bargeton B, Abuin L, Silbering AF, et al. Evolution of acid-sensing olfactory circuits in drosophilids. Neuron. 2017. 10.1016/j.neuron.2016.12.024 28111079

[62] Pitts RJ, Derryberry SL, Zhang Z, Zwiebel LJ. Variant ionotropic receptors in the malaria vector mosquito *Anopheles gambiae* tuned to amines and carboxylic acids. Sci Rep. 2017;7:1–11. 10.1038/srep40297 PMC5220300 28067294

[63] Diehl PA, Vlimant M, Guerenstein P, Guerin PM. Ultrastructure and receptor cell responses of the antennal grooved peg sensilla of *Triatoma infestans* (Hemiptera: Reduviidae). Arthropod Struct Dev. 2003;31:271–85. 10.1016/S1467-8039(03)00004-5 18088986

[64] Guerenstein, PG. Sensory and behavioural responses of *Triatoma infestans* to host and conspecific odours. Switzerland: Ph.D. Thesis, University of Neuchâtel; 1999. pp. 137.

[65] Bernard J. Étude électrophysiologique de récepteurs impliqués dans l’orientation vers l’hôte et dans l’acte hématophage chez un Hémiptère: *Triatoma infestans*. France: Ph.D. Thesis, University of Rennes; 1974. pp. 285.

[66] Taneja J, Guerin PM. Ammonia attracts the haematophagous bug *Triatoma infestans*: behavioural and neurophysiological data on nymphs. J Comp Physiol A. 1997;181:21–34.

[67] Taneja J, Guerin PM. Oriented responses of the triatomine bugs *Rhodnius prolixus* and *Triatoma infestans* to vertebrate odours on a servosphere. J Comp Physiol A. 1995.

[68] Raji JI, Melo N, Castillo JS, Gonzalez S, Saldana V, Stensmyr MC, et al. *Aedes aegypti* mosquitoes detect acidic volatiles found in human odor using the Ir8a pathway. Curr Biol. 2019;29:1253–62. 10.1016/j.cub.2019.02.045 PMC6482070 30930038

[69] Lazzari CR, Nunez JA. Blood Temperature and feeding behavior in *Triatoma infestans* (Heteroptera: Reduviidae). Entomol Gener. 1989;14:183–8.

[70] Ni L, Klein M, Svec KV, Budelli G, Change EC, Ferrer Aj. The ionotropic receptors *Ir21a* and *Ir25a* mediate cool sensing in *Drosophila*. eLife. 2016;5:e13254. 10.7554/eLife.13254 PMC4851551 27126188

[71] Wigglesworth BYVB, Gillett JD. The Function of the Antennae in *Rhodnius prolixus* (Hemiptera) and the Mechanism of Orientation to the Host. J Exp Biol. 1934;11:120–39.

[72] Brown AW (1966). The attraction of mosquitoes to hosts. JAMA.

[73] Enjin A, Zaharieva EE, Frank DD, Mansourian S, Suh GS, Gallio M, et al. Humidity sensing in *Drosophila*. Curr Biol. 2016;26:1352–8. 10.1016/j.cub.2016.03.049 PMC5305172 27161501

[74] Knecht ZA, Silbering AF, Cruz J, Yang L, Croset V, Benton R, et al. Ionotropic Receptor-dependent moist and dry cells control hygrosensation in *Drosophila*. Elife. 2017;6:e26654. 10.7554/eLife.26654 PMC5495567 28621663

[75] Knecht ZA, Silbering AF, Ni L, Klein M, Budelli G, Bell R, et al. Distinct combinations of variant ionotropic glutamate receptors mediate thermosensation and hygrosensation in *Drosophila*. Elife. 2016;5:e17879. 10.7554/eLife.17879 PMC5052030 27656904

[76] Touhara K, Prestwich GD (1992). Binding site mapping of a photoaffinity-labeled juvenile hormone binding protein. Biochem Biophys Res Commun.

[77] Sarov-blat L, So WV, Liu L, Rosbash M, Hughes H. The *Drosophila takeout* gene is a novel molecular link between circadian rhythms and feeding behavior. Cell. 2000;101:647–56. 10.1016/s0092-8674(00)80876-4 10892651

[78] Meunier N, Belgacem YH, Martin J. Regulation of feeding behaviour and locomotor activity by takeout in *Drosophila*. J Exp Biol. 2007;210(Pt 8):1424–34. 10.1242/jeb.02755 17401125

[79] Du J, Hiruma K, Riddiford LM. A novel gene in the *takeout* gene family is regulated by hormones and nutrients in *Manduca *larval epidermis. Insect Biochem Mol Biol. 2003;33:803–14. 10.1016/s0965-1748(03)00079-1 12878227

[80] Poivet E, Gallot A, Montagné N, Senin P, Monsempès C, Legeai F, et al. Transcriptome profiling of starvation in the peripheral chemosensory organs of the crop pest *Spodoptera littoralis* caterpillars. Insects. 2021;12:573. 10.3390/insects12070573 PMC8303696 34201462

[81] Albone ES. Mammalian Semiochemistry. The investigation of chemical signals between mammals. Chichester: Wiley: 1984.

[82] Norwood DM, Wainman T, Lioy PJ, Waldman JM (1992). Breath ammonia depletion and its relevance to acidic aerosol exposure studies. Arch Environ Health Int J.

[83] Reisenman CE. Hunger is the best spice: Effects of starvation in the antennal responses of the blood-sucking bug *Rhodnius prolixus*. J Insect Physiol. 2014;71:8–13. 10.1016/j.jinsphys.2014.09.009 PMC4258481 25280630

[84] Vulpe A, Kim HS, Ballou S, Wu S-T, Grabe V, Gonzales CN (2021). An ammonium transporter is a non-canonical olfactory receptor for ammonia. Curr Biol.

[85] Menuz K, Larter NK, Park J, Carlson JR. An RNA-Seq screen of the *Drosophila a*ntenna identifies a transporter necessary for ammonia detection. PLoS Genet. 2014;10(11):e1004810. 10.1371/journal.pgen.1004810 PMC4238959 25412082

[86] Pitts RJ, Derryberry SL, Pulous FE, Zwiebel LJ. Antennal-expressed ammonium transporters in the malaria vector mosquito *Anopheles gambiae*. PLoS ONE. 2014;9(10):e111858. 10.1371/journal.pone.0111858 PMC4216128 25360676

[87] McDonald TR, Ward JM (2016). Evolution of electrogenic ammonium transporters (AMTs). Front Plant Sci.

[88] Hill SR, Ghaninia M, Ignell R. Blood meal induced regulation of gene expression in the maxillary palps, a chemosensory organ of the mosquito *Aedes aegypti*. Front Ecol Evol. 2019;7:336.

[89] Sánchez-Gracia A, Vieira FG, Rozas J (2009). Molecular evolution of the major chemosensory gene families in insects. Heredity.

[90] Liu F, Chen Z, Liu N. Molecular basis of olfactory chemoreception in the common bed bug *Cimex lectularius. *Sci Rep. 2017;7:45531. 10.1038/srep45531 PMC5382537 28383033

[91] Liu F, Liu N. Human odorant reception in the common bed gug *Cimex lectularius. *Sci Rep. 2015;5:1–14. 10.1038/srep15558 PMC4629130 26522967

[92] Matthews BJ, Dudchenko O, Kingan SB, Koren S, Antoshechkin I, Crawford JE, et al. Improved reference genome of *Aedes aegypti* informs arbovirus vector control. Nature. 2018;563:501–7. 10.1038/s41586-018-0692-z PMC6421076 30429615

[93] Robertson HM, Baits RL, Walden KKO, Wada-Katsumata A, Schal C. Enormous expansion of the chemosensory gene repertoire in the omnivorous German cockroach *Blattella germanica*. J Exp Zoolog B Mol Dev Evol. 2018;330:265–78. 10.1002/jez.b.22797 PMC6175461 29566459

[94] Terrapon N, Li C, Robertson HM, Ji L, Meng X, Booth W (2014). Molecular traces of alternative social organization in a termite genome. Nat Commun.

[95] Pelosi P, Iovinella I, Zhu J, Wang G, Dani FR. Beyond chemoreception: diverse tasks of soluble olfactory proteins in insects. Biol Rev. 2018. 10.1111/brv.12339 28480618

[96] Fowler MA, Montell C. *Drosophila *TRP channels and animal behavior. Life Sci. 2013;92:394–403. 10.1016/j.lfs.2012.07.029 PMC3524398 22877650

[97] Badsha F, Kain P, Prabhakar S, Sundaram S, Padinjat R, Rodrigues V, et al. Mutants in *Drosophila *TRPC channels reduce olfactory sensitivity to carbon dioxide. PLoS ONE. 2012;7:e49848. 10.1371/journal.pone.0049848 PMC3501451 23185459

[98] Latorre-Estivalis JM, Almeida FC, Pontes G, Dopazo H, Barrozo RB, Lorenzo MG (2021). Evolution of the insect PPK gene family. Genome Biol Evol..

[99] Vanaphan N, Dauwalder B, Zufall RA. Diversification of *takeout*, a male-biased gene family in *Drosophila*. Gene. 2012;491:142–8. 10.1016/j.gene.2011.10.003 22020223

[100] Kim HG, Margolies D, Park Y. The roles of thermal transient receptor potential channels in thermotactic behavior and in thermal acclimation in the red flour beetle. *Tribolium castaneum.* J Insect Physiol. 2015;76:47–55. 10.1016/j.jinsphys.2015.03.008 25813190

[101] Rosenzweig M, Brennan KM, Tayler TD, Phelps PO, Patapoutian A, Garrity PA. The *Drosophila *ortholog of vertebrate TRPA1 regulates thermotaxis. Genes Dev. 2005;19:419–24. 10.1101/gad.1278205 PMC548941 15681611

[102] Cassau S, Krieger J (2021). The role of SNMPs in insect olfaction. Cell Tissue Res.

[103] Zhang Y-N, Zhu X-Y, Zhang Q, Yin C-Y, Dong Z-P, Zuo L-H, et al. De novo assembly and characterization of antennal transcriptome reveal chemosensory system in *Nysius ericae*. J Asia-Pac Entomol. 2016;19:1077–87.

[104] Tanaka K, Shimomura K, Hosoi A, Sato Y, Oikawa Y, Seino Y, et al. Antennal transcriptome analysis of chemosensory genes in the cowpea beetle (F.) *Callosobruchus maculatus*. Plos One. 2022;17:e0262817. 10.1371/journal.pone.0262817 PMC8769365 35045135

[105] An XK, Sun L, Liu HW, Liu DF, Ding YX, Li LM, et al. Identification and expression analysis of an olfactory receptor gene family in green plant bug *Apolygus lucorum* (Meyer-Dür). Sci Rep. 2016;6:37870. 10.1038/srep37870 PMC5124970 27892490

[106] Hordijk W, Gascuel O. Improving the efficiency of SPR moves in phylogenetic tree search methods based on maximum likelihood. Bioinformatics. 2005;21(24):4338–47. 10.1093/bioinformatics/bti713 16234323

[107] Minh BQ, Nguyen MAT, von Haeseler A. Ultrafast approximation for phylogenetic bootstrap. Mol Biol Evol. 2013;30(5):1188–95. 10.1093/molbev/mst024 PMC3670741 23418397

[108] Zhu A, Ibrahim JG, Love MI (2019). Heavy-tailed prior distributions for sequence count data: removing the noise and preserving large differences. Bioinformatics.

[109] Lee HK, Braynen W, Keshav K, Pavlidis P (2005). ErmineJ: tool for functional analysis of gene expression data sets. BMC Bioinformatics.

[110] Zelle KM, Lu B, Pyfrom SC, Ben-Shahar Y. The genetic architecture of degenerin/epithelial sodium channels in *Drosophila*. G3 Genes Genomes Genet. 2013;3:441–50. 10.1534/g3.112.005272 PMC3583452 23449991

[111] Torres-Oliva M, Almeida FC, Sánchez-Gracia A, Rozas J (2016). Comparative genomics uncovers unique gene turnover and evolutionary rates in a gene family involved in the detection of insect cuticular pheromones. Genome Biol Evol.

