# Supplementary figures and images for: The antennal transcriptome of Triatoma infestans reveals substantial expression changes triggered by a blood meal
Source: BMC Genomics. 2022 Dec 30;23:861. doi: 10.1186/s12864-022-09059-6 (PMC9801554; doi:10.1186/s12864-022-09059-6)

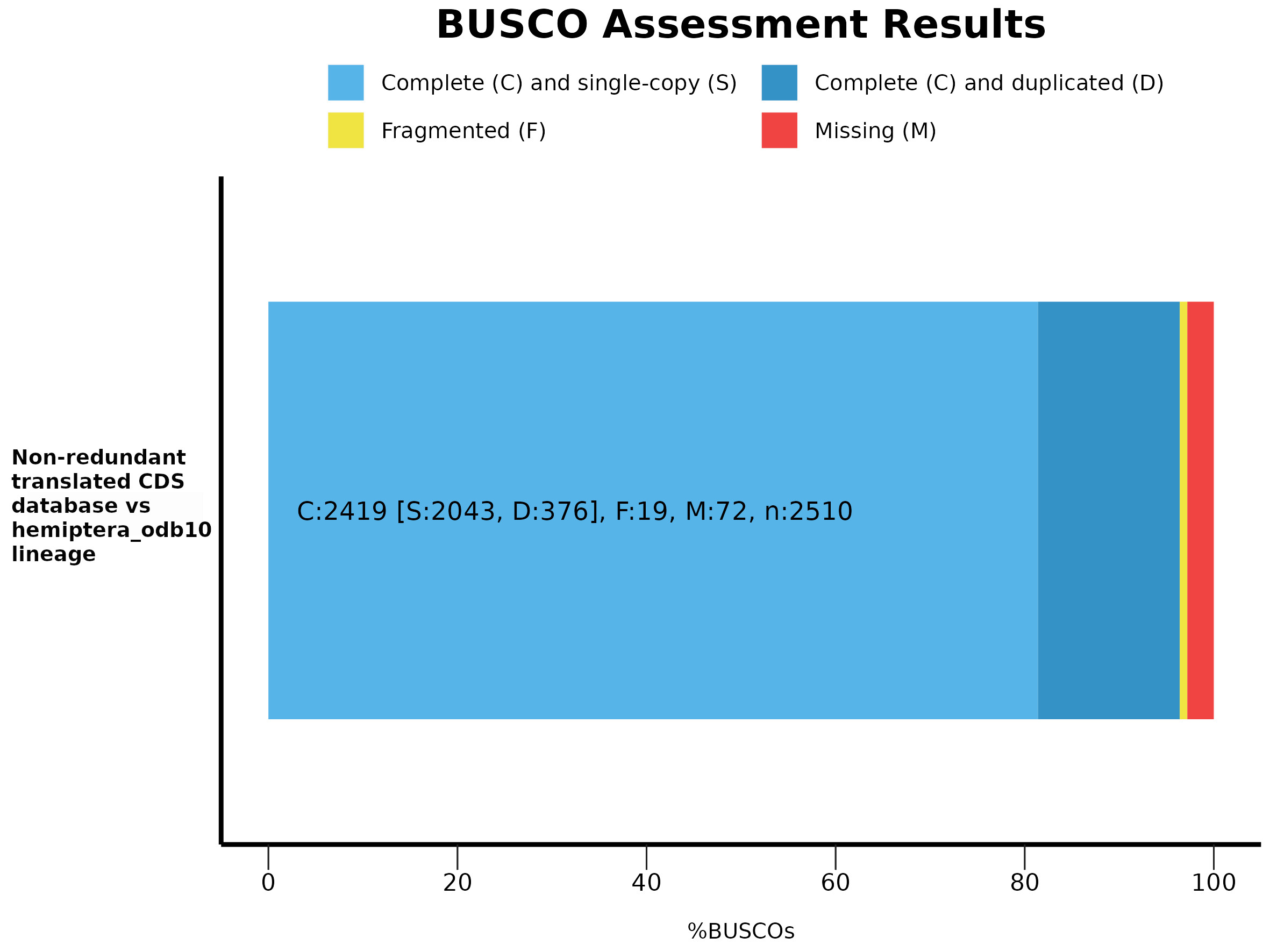

Supplement: Supplementary file 2 — Additional file 2: Supplementary Figure S1. Graphical summary of the BUSCO assessment results of the non-redundant translated CDS database against Hemiptera. [file 12864_2022_9059_MOESM2_ESM.tif]

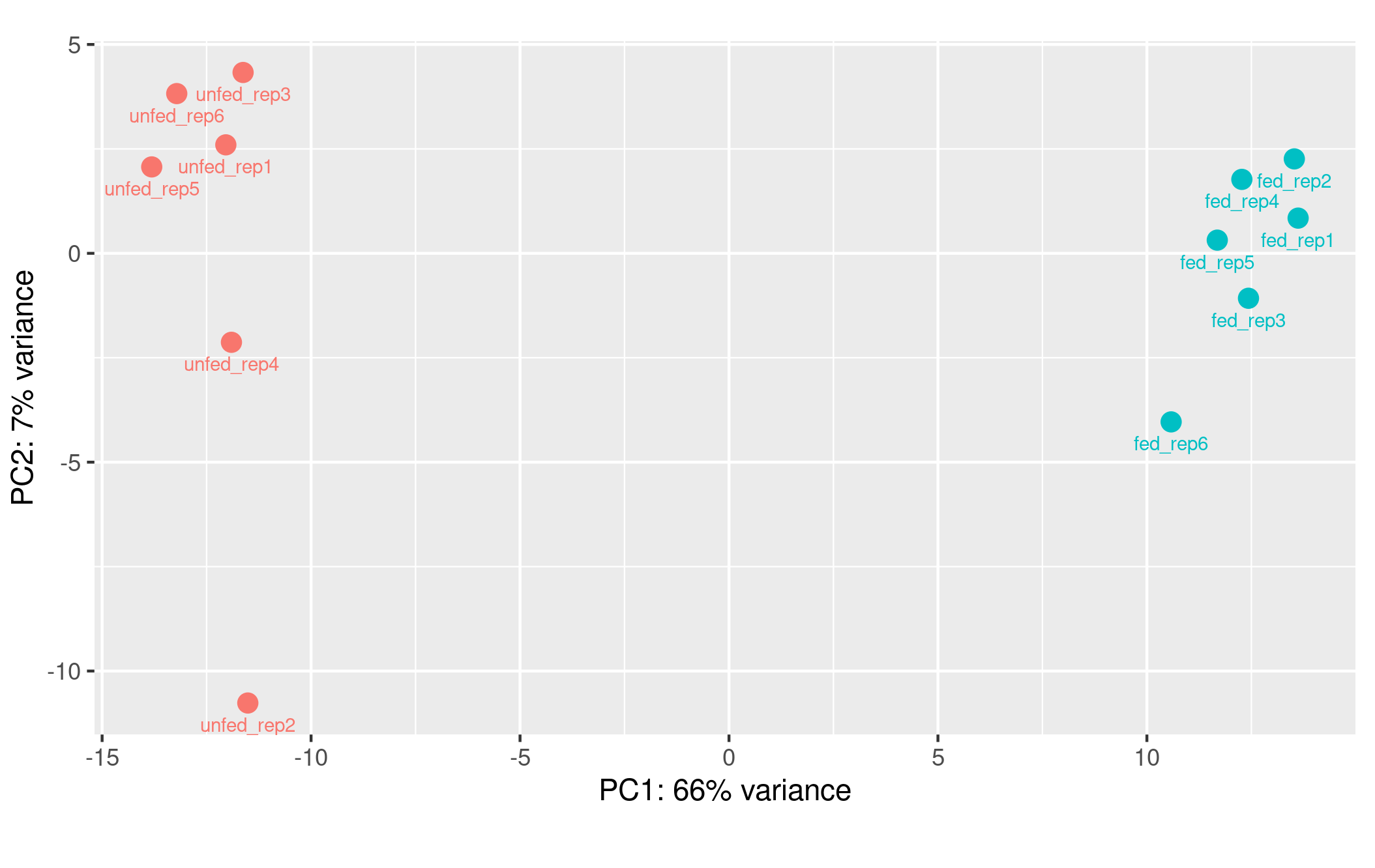

Supplement: Supplementary file 9 — Additional file 9: Supplementary Figure S4. Principal Component Analysis graph. [file 12864_2022_9059_MOESM9_ESM.tif]
