# Supplementary material for: The antennal transcriptome of Triatoma infestans reveals substantial expression changes triggered by a blood meal
Source: BMC Genomics. 2022 Dec 30;23:861. doi: 10.1186/s12864-022-09059-6 (PMC9801554; doi:10.1186/s12864-022-09059-6)
Supplement: Supplementary file 7 — Additional file 7: Supplementary Figure S3. Transcript abundance of the T. infestans sensory-related gene families. OR (A), IR (B), GR (C), OBP (D), CSP (E), TO (F), TRP (G), PPK (H) SNMP (I), ammonium transporter (J) and CHE (K) heatmaps were created using Log10 (Transcripts Per kilobase per Million reads +1) as input of the gplot R package. Transcript abundance was represented in a color scale where white/red represents the lowest/highest expression. A dendrogram was plotted using hierarchical clustering of genes based on euclidean distances and a complete linkage method for clustering. UF: unfed bug samples (control) and F: fed bug samples. [file 12864_2022_9059_MOESM7_ESM.pdf]

Additional file 7: Supplementary Figure S3. Transcript abundance of the T. infestans sensory-related gene families. OR (A), IR (B), GR (C), OBP (D), CSP (E), TO (F), TRP (G), PPK (H) and SNMP (I), ammonium transporter (J) and CHE (K)

A) ORs

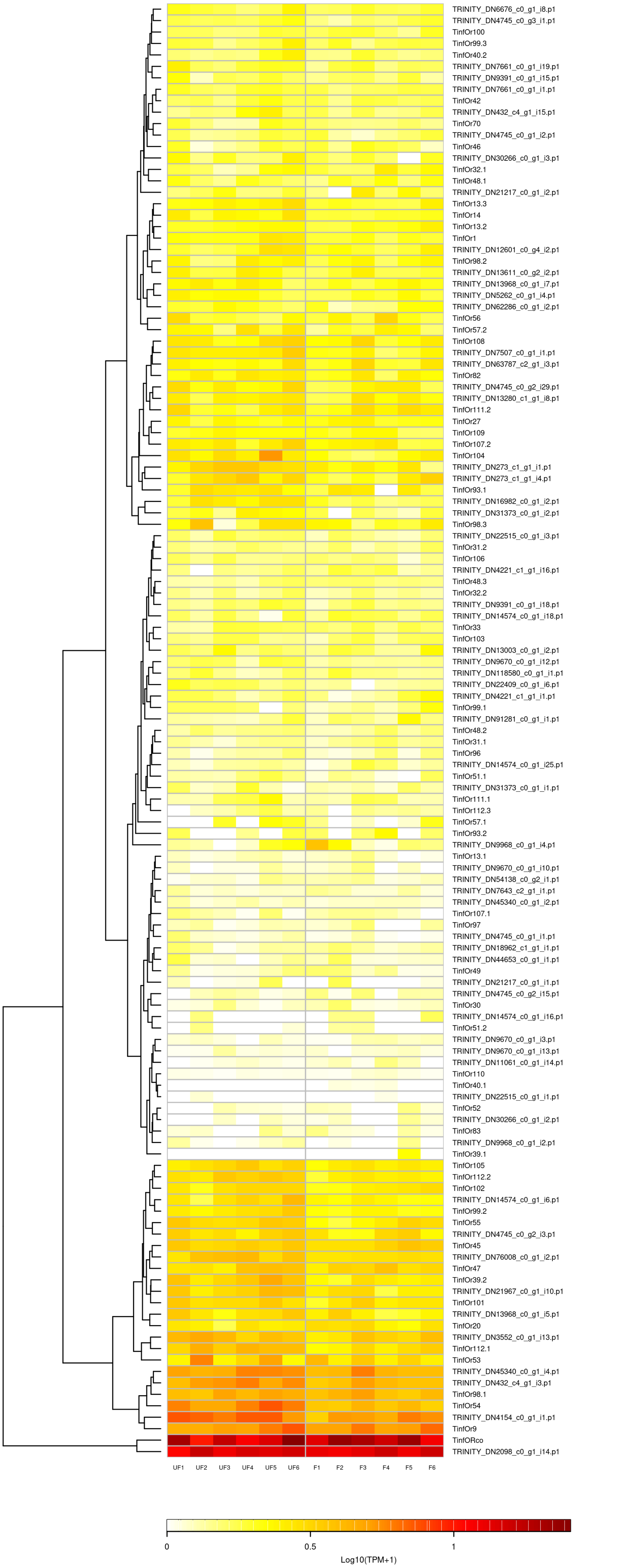

B) IRs

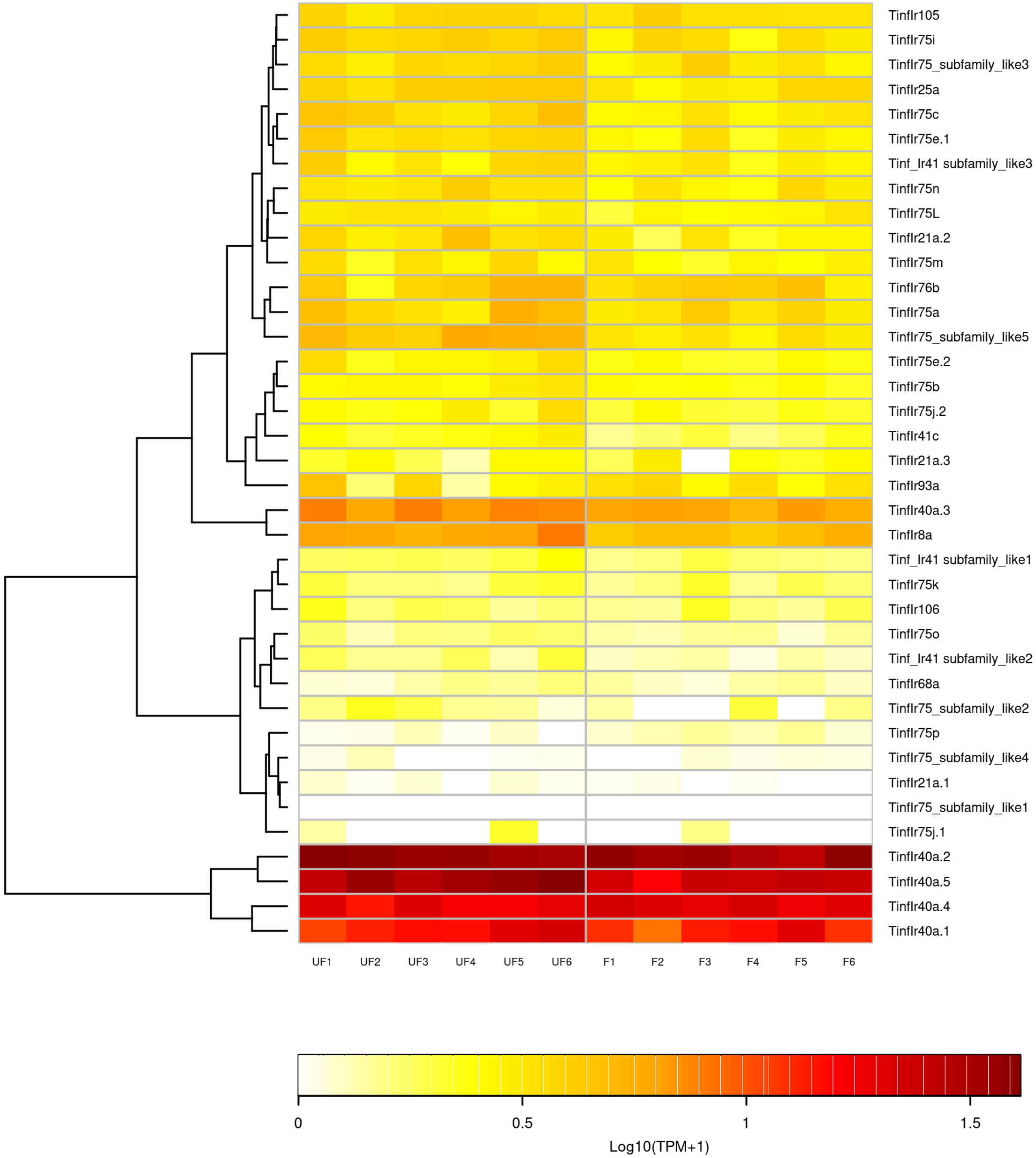

TinfIr105
TinfIr75i
TinfIr75_subfamily_like3
TinfIr25a
TinfIr75c
TinfIr75e.1
Tinf_Ir41 subfamily_like3
TinfIr75n
TinfIr75L
TinfIr21a.2
TinfIr75m
TinfIr76b
TinfIr75a
TinfIr75_subfamily_like5
TinfIr75e.2
TinfIr75b
TinfIr75j.2
TinfIr41c
TinfIr21a.3
TinfIr93a
TinfIr40a.3
TinfIr8a
Tinf_Ir41 subfamily_like1
TinfIr75k
TinfIr106
TinfIr75o
Tinf_Ir41 subfamily_like2
TinfIr68a
TinfIr75_subfamily_like2
TinfIr75p
TinfIr75_subfamily_like4
TinfIr21a.1
TinfIr75_subfamily_like1
TinfIr75j.1
TinfIr40a.2
TinfIr40a.5
TinfIr40a.4
TinfIr40a.1
UF1
UF2
UF3
UF4
UF5
UF6
F1
F2
F3
F4
F5
F6
0
0.5
1
1.5
Log10(TPM+1)

C) GRs

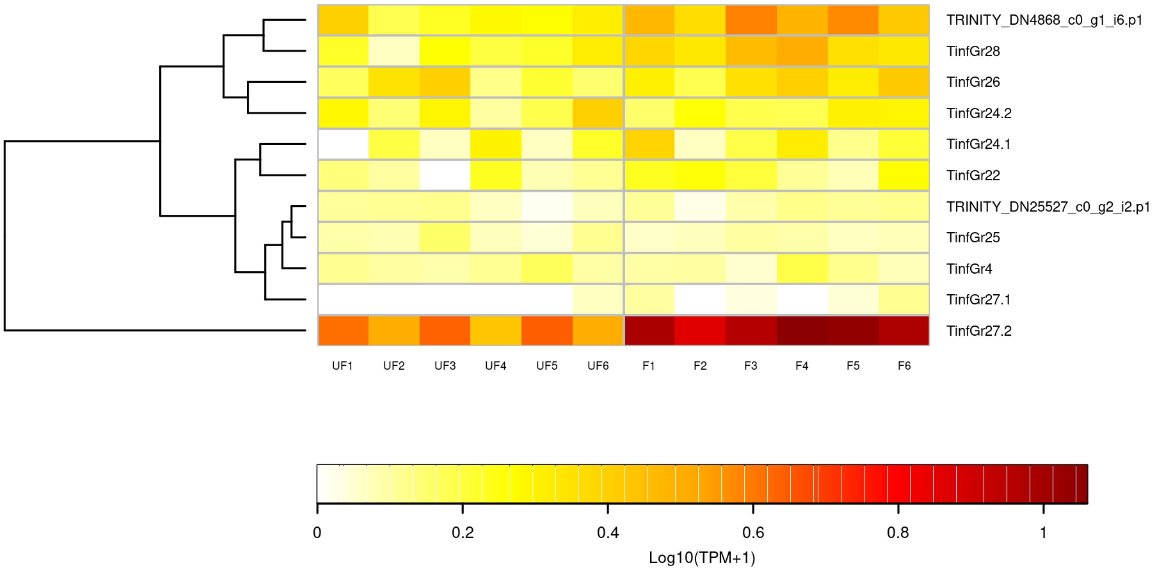
TRINITY_DN4868_c0_g1_i6.p1
TinfGr28
TinfGr26
TinfGr24.2
TinfGr24.1
TinfGr22
TRINITY_DN25527_c0_g2_i2.p1
TinfGr25
TinfGr4
TinfGr27.1
TinfGr27.2
UF1
UF2
UF3
UF4
UF5
UF6
F1
F2
F3
F4
F5
F6
0
0.2
0.4
0.6
0.8
1
Log10(TPM+1)

D) OBPs

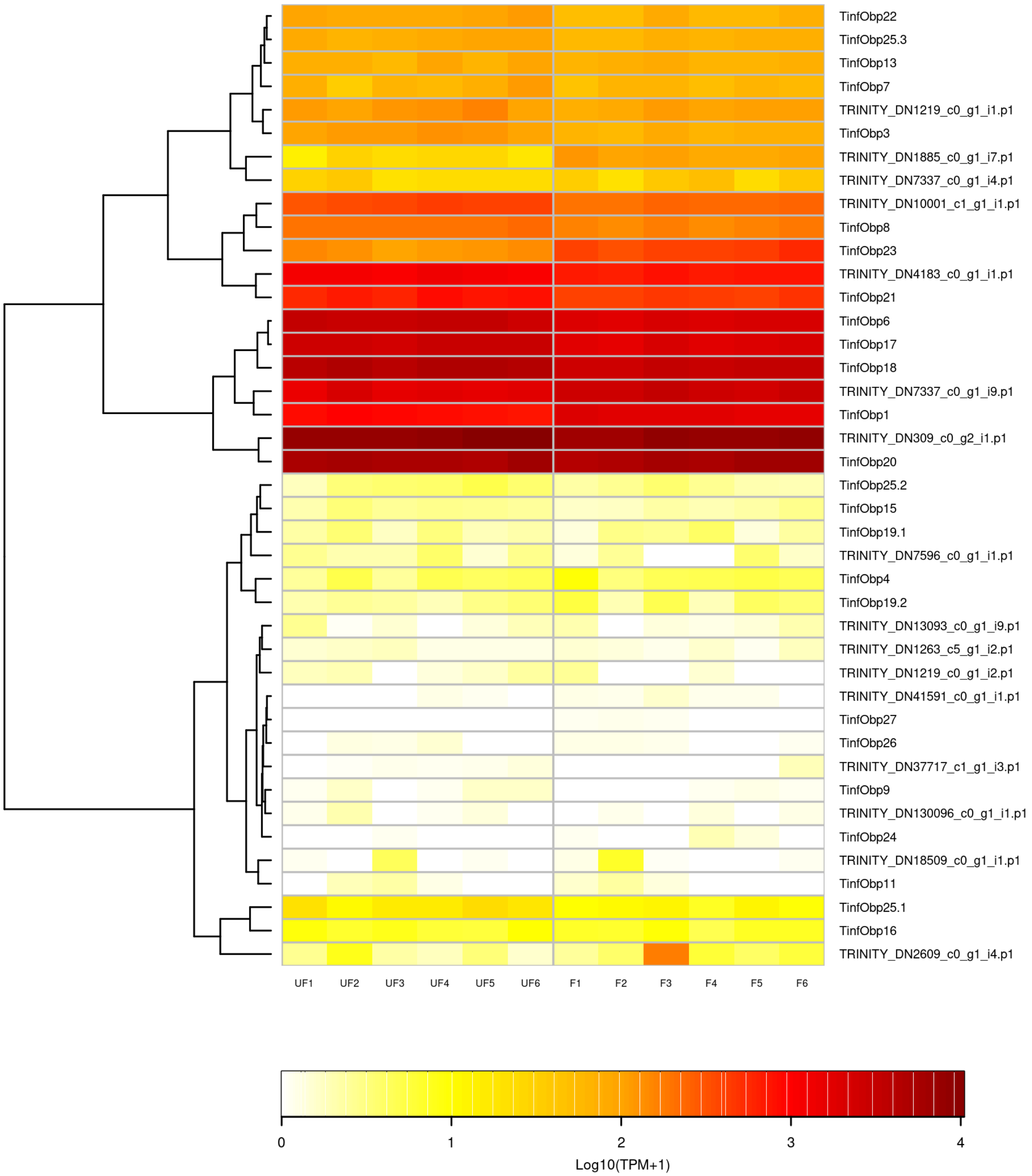

TinfObp22
TinfObp25.3
TinfObp13
TinfObp7
TRINITY_DN1219_c0_g1_i1.p1
TinfObp3
TRINITY_DN1885_c0_g1_i7.p1
TRINITY_DN7337_c0_g1_i4.p1
TRINITY_DN10001_c1_g1_i1.p1
TinfObp8
TinfObp23
TRINITY_DN4183_c0_g1_i1.p1
TinfObp21
TinfObp6
TinfObp17
TinfObp18
TRINITY_DN7337_c0_g1_i9.p1
TinfObp1
TRINITY_DN309_c0_g2_i1.p1
TinfObp20
TinfObp25.2
TinfObp15
TinfObp19.1
TRINITY_DN7596_c0_g1_i1.p1
TinfObp4
TinfObp19.2
TRINITY_DN13093_c0_g1_i9.p1
TRINITY_DN1263_c5_g1_i2.p1
TRINITY_DN1219_c0_g1_i2.p1
TRINITY_DN41591_c0_g1_i1.p1
TinfObp27
TinfObp26
TRINITY_DN37717_c1_g1_i3.p1
TinfObp9
TRINITY_DN130096_c0_g1_i1.p1
TinfObp24
TRINITY_DN18509_c0_g1_i1.p1
TinfObp11
TinfObp25.1
TinfObp16
TRINITY_DN2609_c0_g1_i4.p1
UF1
UF2
UF3
UF4
UF5
UF6
F1
F2
F3
F4
F5
F6
0
1
2
3
4
Log10(TPM+1)

E) CSPs

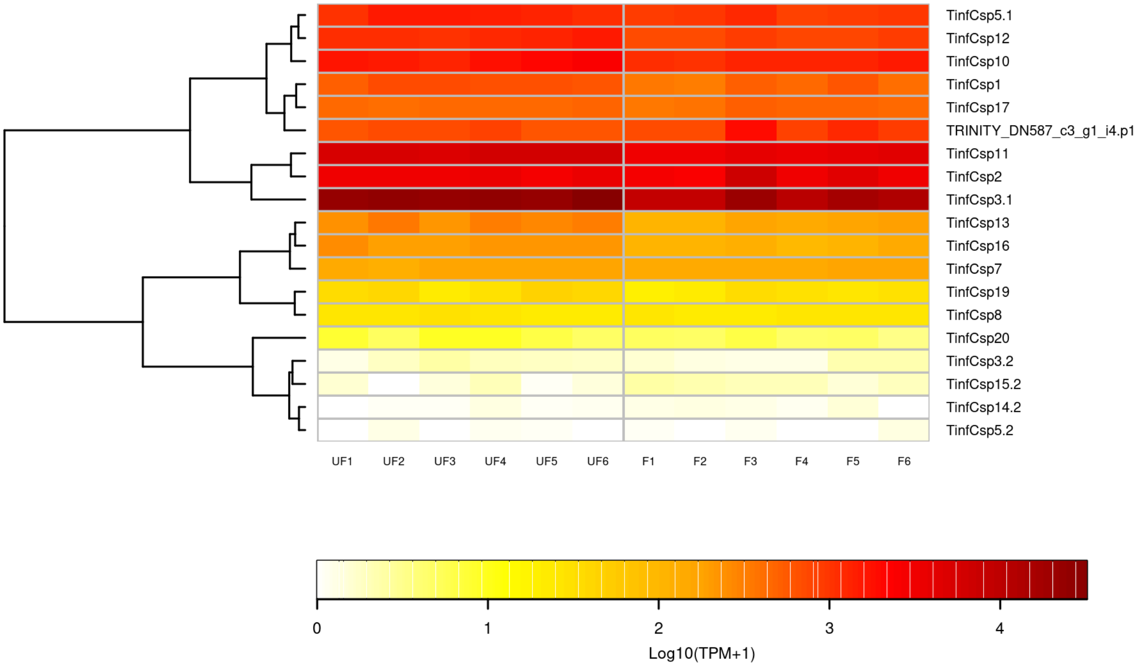
TinfCsp5.1
TinfCsp12
TinfCsp10
TinfCsp1
TinfCsp17
TRINITY_DN587_c3_g1_i4.p1
TinfCsp11
TinfCsp2
TinfCsp3.1
TinfCsp13
TinfCsp16
TinfCsp7
TinfCsp19
TinfCsp8
TinfCsp20
TinfCsp3.2
TinfCsp15.2
TinfCsp14.2
TinfCsp5.2
UF1
UF2
UF3
UF4
UF5
UF6
F1
F2
F3
F4
F5
F6
0
1
2
3
4
Log10(TPM+1)

F) TOs

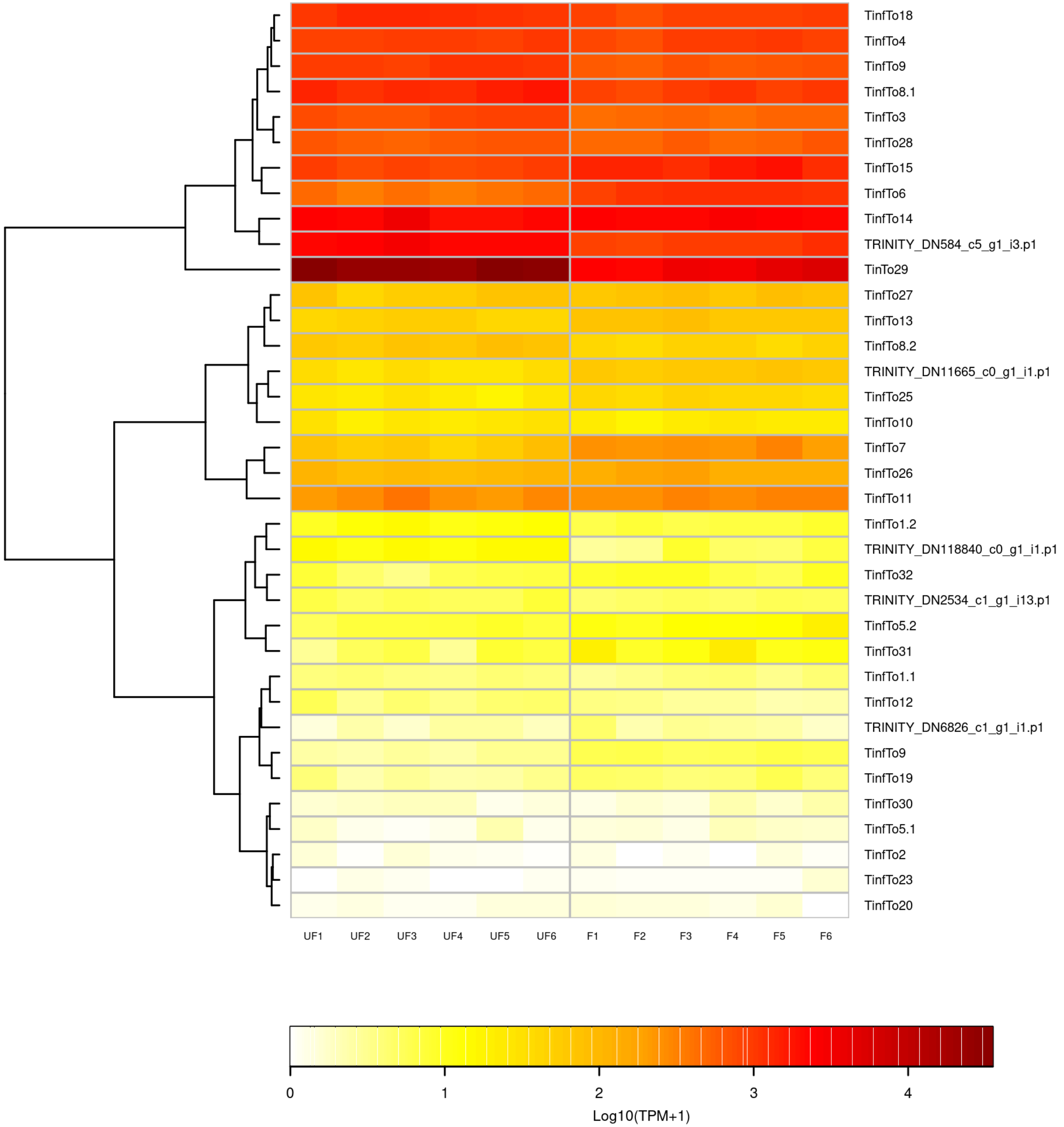
TinfTo18
TinfTo4
TinfTo9
TinfTo8.1
TinfTo3
TinfTo28
TinfTo15
TinfTo6
TinfTo14
TRINITY_DN584_c5_g1_i3.p1
TinTo29
TinfTo27
TinfTo13
TinfTo8.2
TRINITY_DN11665_c0_g1_i1.p1
TinfTo25
TinfTo10
TinfTo7
TinfTo26
TinfTo11
TinfTo1.2
TRINITY_DN118840_c0_g1_i1.p1
TinfTo32
TRINITY_DN2534_c1_g1_i13.p1
TinfTo5.2
TinfTo31
TinfTo1.1
TinfTo12
TRINITY_DN6826_c1_g1_i1.p1
TinfTo9
TinfTo19
TinfTo30
TinfTo5.1
TinfTo2
TinfTo23
TinfTo20
UF1
UF2
UF3
UF4
UF5
UF6
F1
F2
F3
F4
F5
F6
0
1
2
3
4
Log10(TPM+1)

G) TRPs

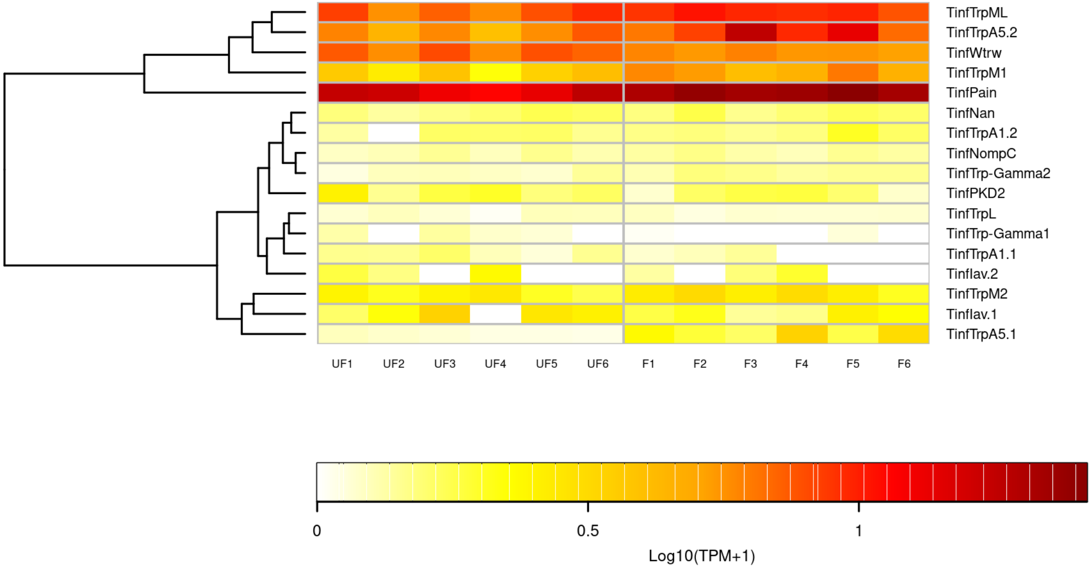

TinfTrpML
TinfTrpA5.2
TinfWtrw
TinfTrpM1
TinfPain
TinfNan
TinfTrpA1.2
TinfNompC
TinfTrp-Gamma2
TinfPKD2
TinfTrpL
TinfTrp-Gamma1
TinfTrpA1.1
Tinflav.2
TinfTrpM2
Tinflav.1
TinfTrpA5.1
UF1
UF2
UF3
UF4
UF5
UF6
F1
F2
F3
F4
F5
F6
0
0.5
1
Log10(TPM+1)

H) PPKs

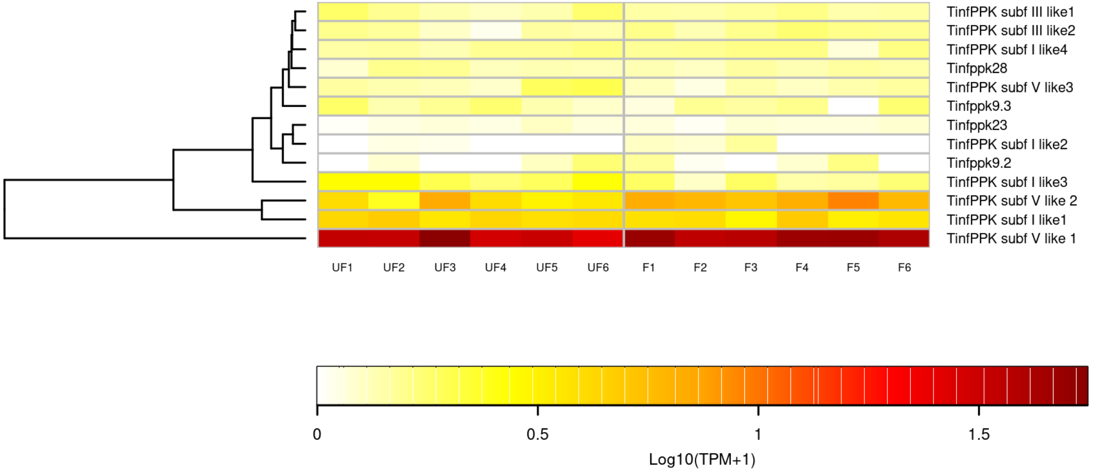

TinfPPK subf III like1
TinfPPK subf III like2
TinfPPK subf I like4
Tinfppk28
TinfPPK subf V like3
Tinfppk9.3
Tinfppk23
TinfPPK subf I like2
Tinfppk9.2
TinfPPK subf I like3
TinfPPK subf V like 2
TinfPPK subf I like1
TinfPPK subf V like 1
UF1
UF2
UF3
UF4
UF5
UF6
F1
F2
F3
F4
F5
F6
0
0.5
1
1.5
Log10(TPM+1)

I) SNMPs

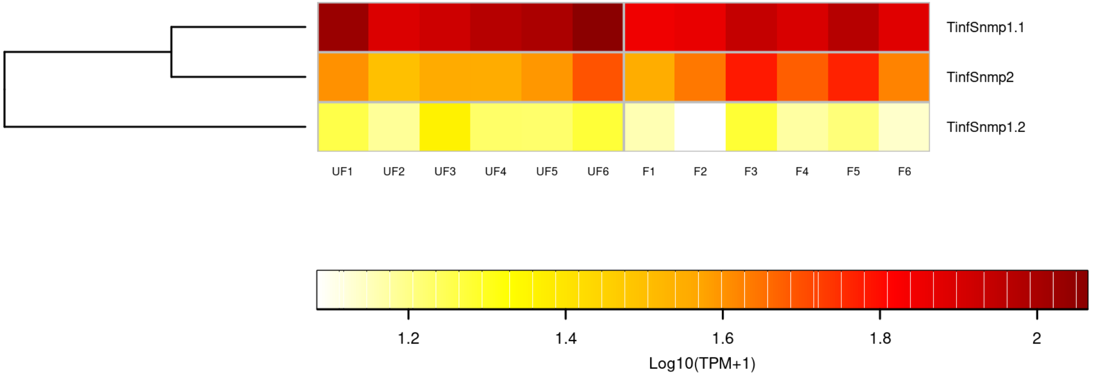
TinfSnmp1.1
TinfSnmp2
TinfSnmp1.2
UF1
UF2
UF3
UF4
UF5
UF6
F1
F2
F3
F4
F5
F6
1.2
1.4
1.6
1.8
2
Log10(TPM+1)

J) AmT

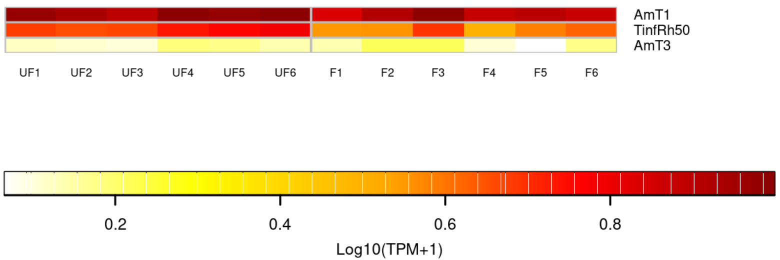
AmT1
TinfRh50
AmT3
UF1
UF2
UF3
UF4
UF5
UF6
F1
F2
F3
F4
F5
F6
0.2
0.4
0.6
0.8
Log10(TPM+1)

K) CHE

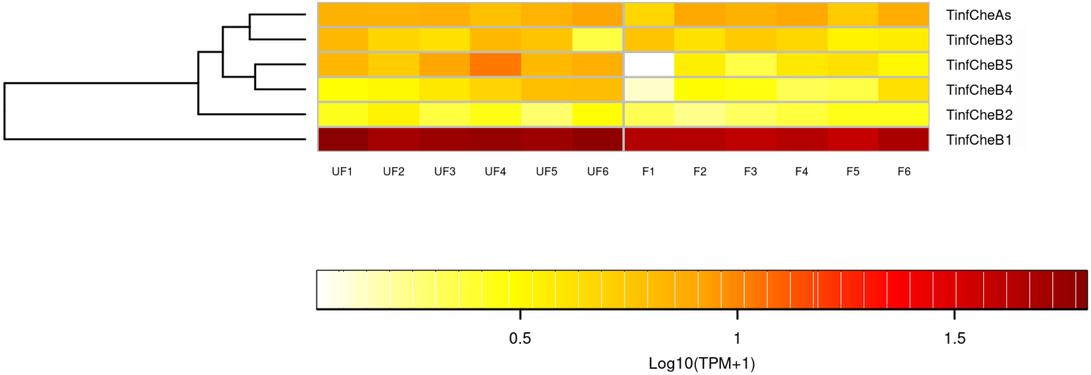
TinfCheAs
TinfCheB3
TinfCheB5
TinfCheB4
TinfCheB2
TinfCheB1
UF1
UF2
UF3
UF4
UF5
UF6
F1
F2
F3
F4
F5
F6
0.5
1
1.5
Log10(TPM+1)
