# Supplementary material for: The antennal transcriptome of Triatoma infestans reveals substantial expression changes triggered by a blood meal
Source: BMC Genomics. 2022 Dec 30;23:861. doi: 10.1186/s12864-022-09059-6 (PMC9801554; doi:10.1186/s12864-022-09059-6)

Additional file 6: Supplementary Figure S2. Phylogenetic trees of sensory-related gene families analyzed.

The maximum-likelihood trees were constructed using IQ-Tree and support values are shown for branches with both aLRT-SH and UFBoot values.

Down and up-regulated transcripts after blood ingestion are marked with blue and red circles in the trees.

A) OR phylogenetic tree

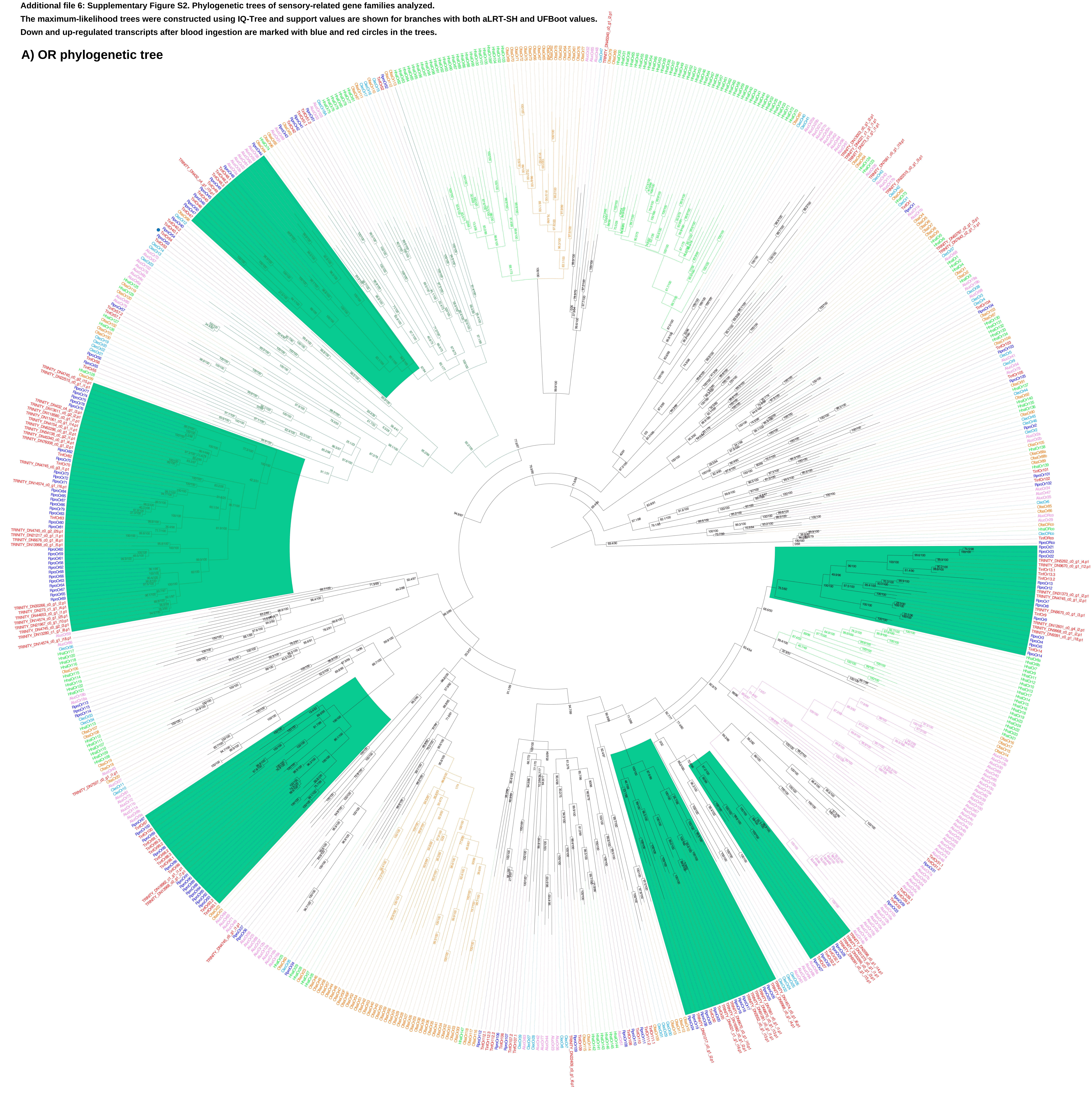

B) GR phylogenetic tree

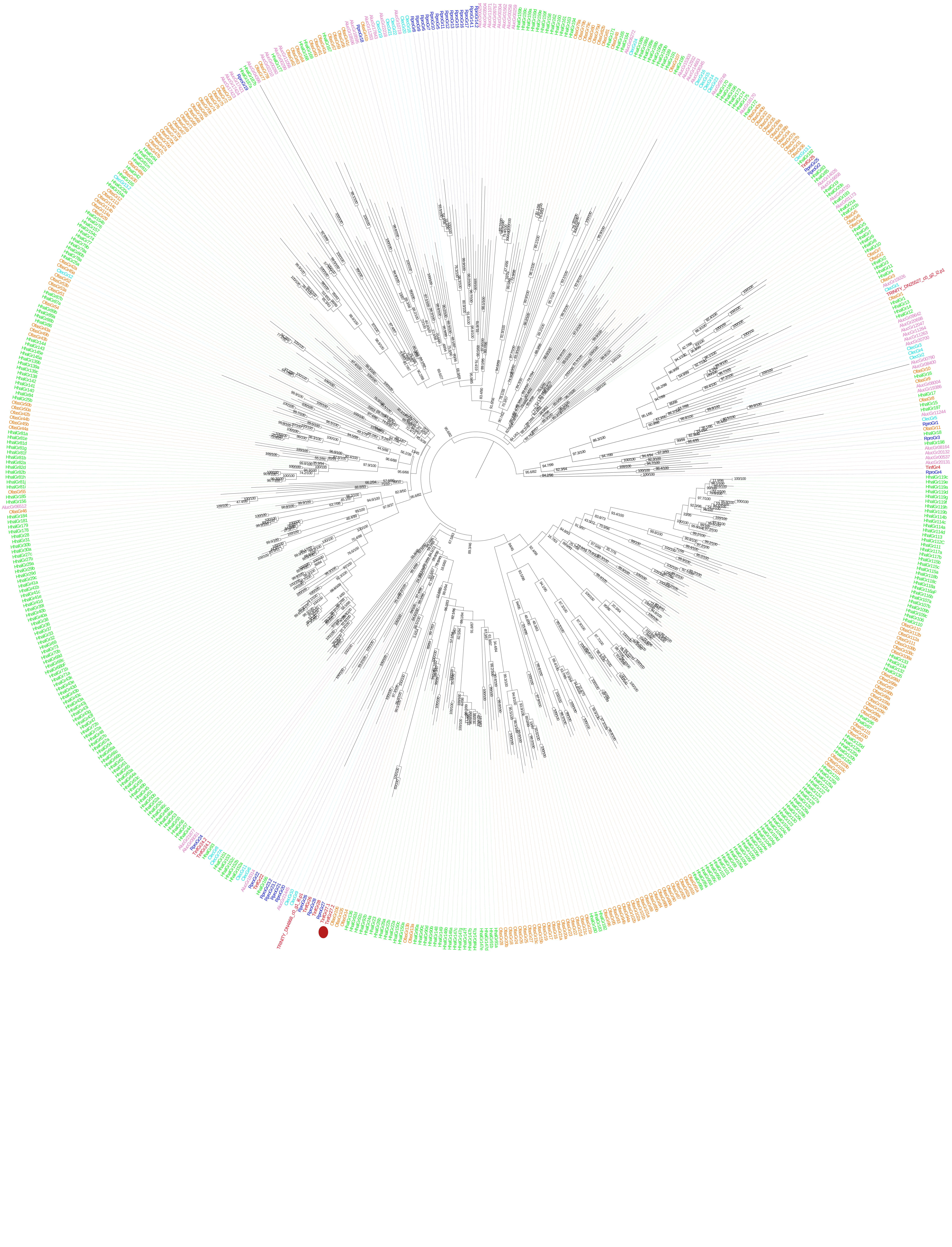

c) TOs phylogenetic tree

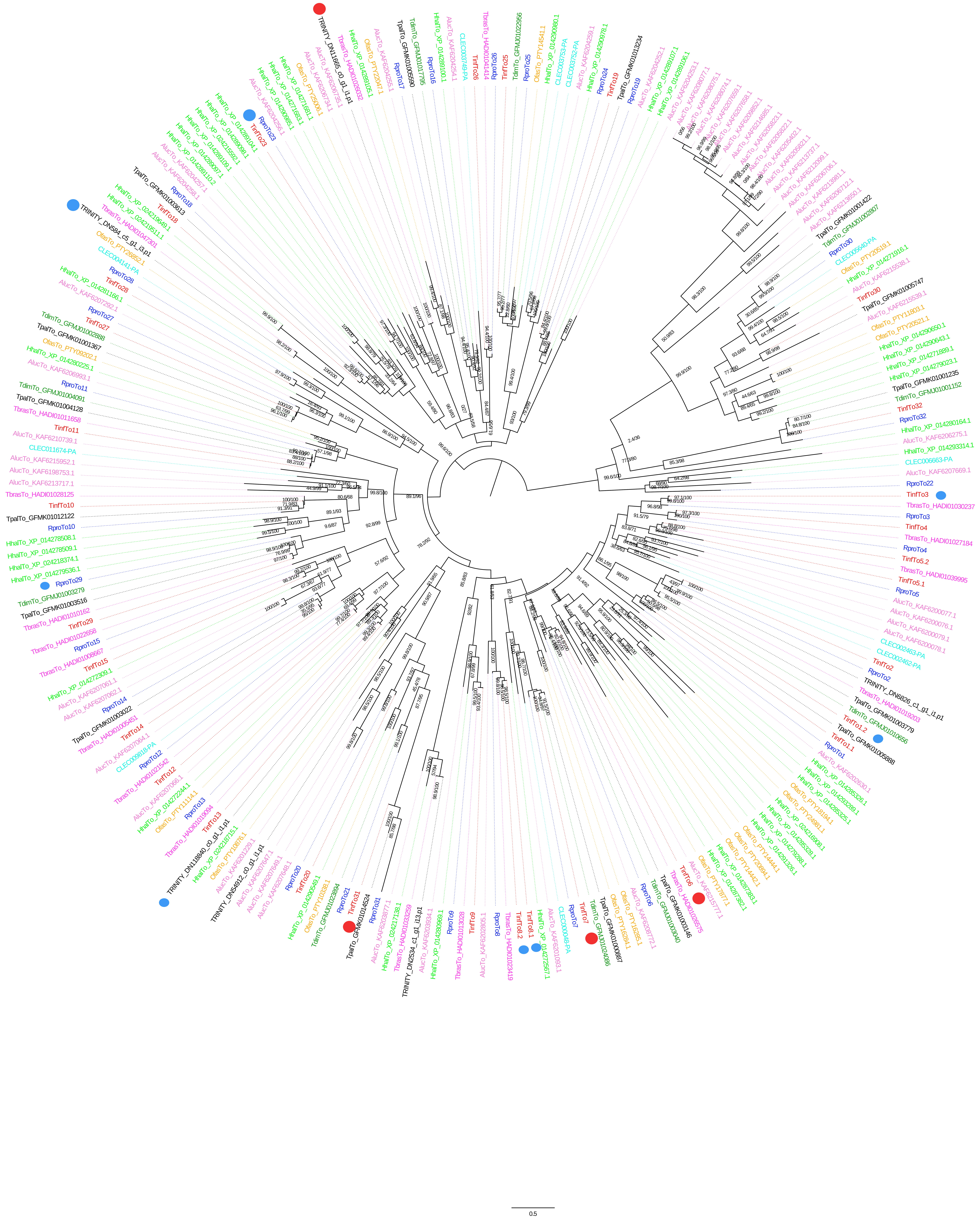

D) TRP phylogenetic tree

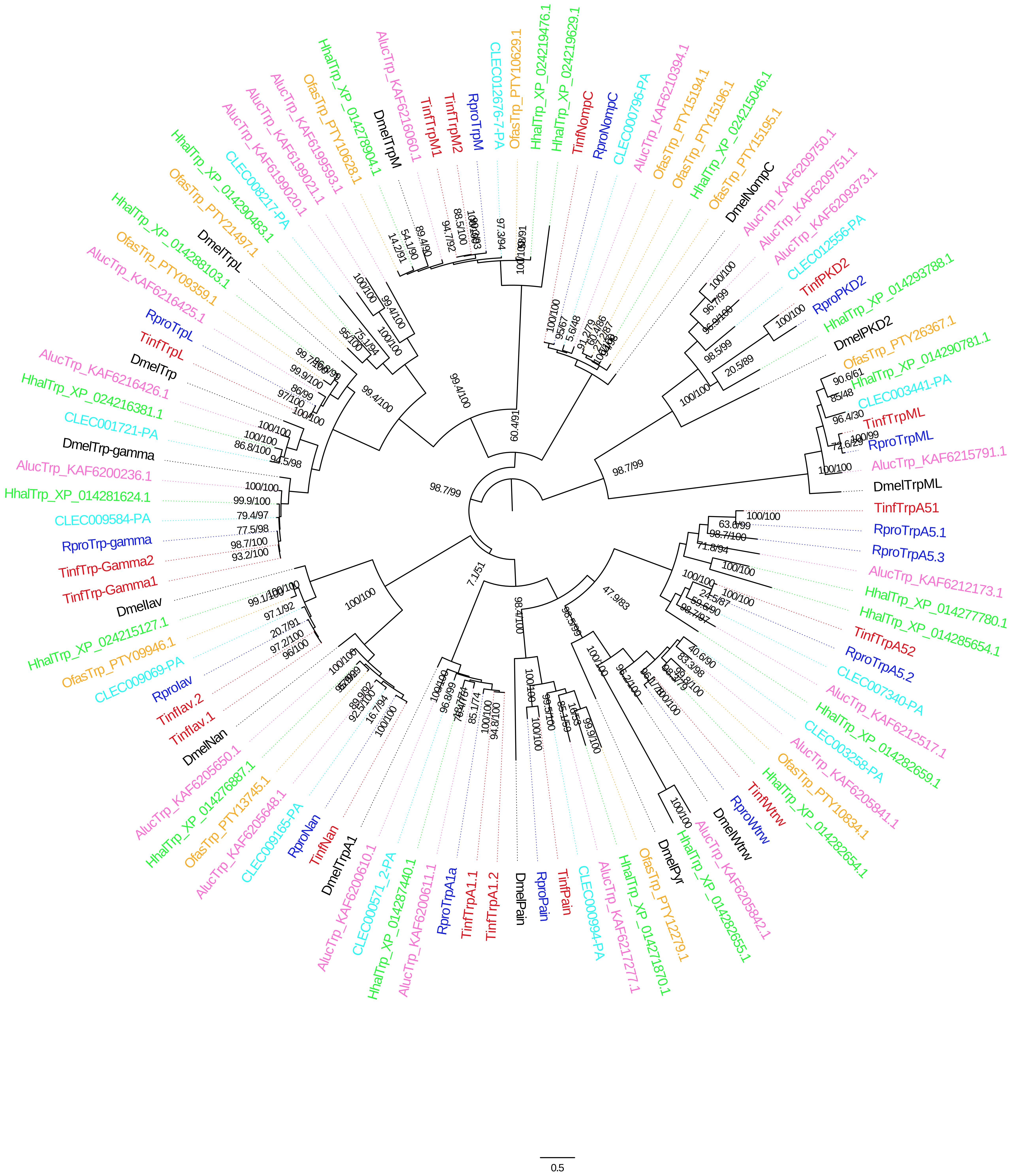

E) PPK phylogenetic tree

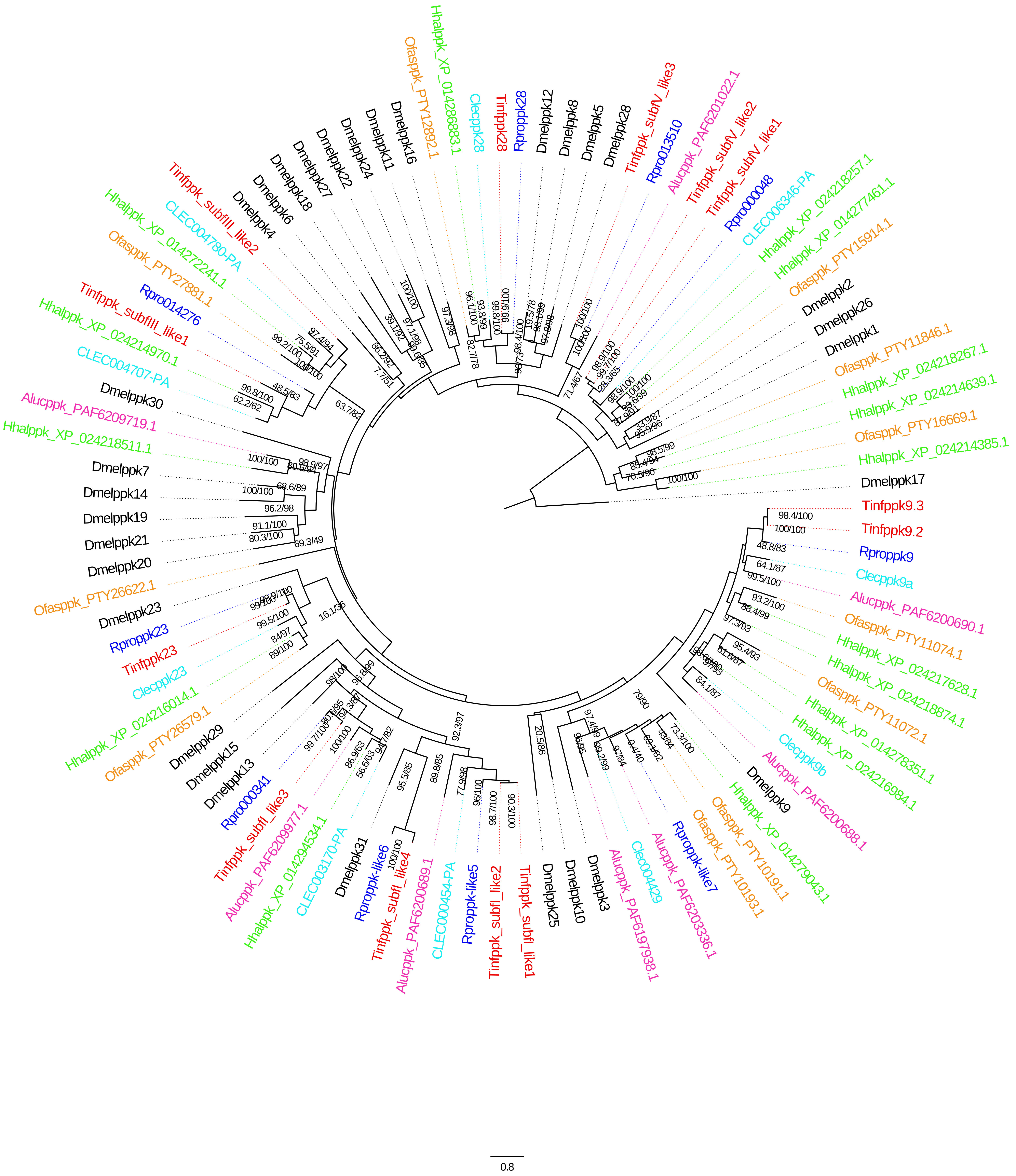

F) SNMP phylogenetic tree

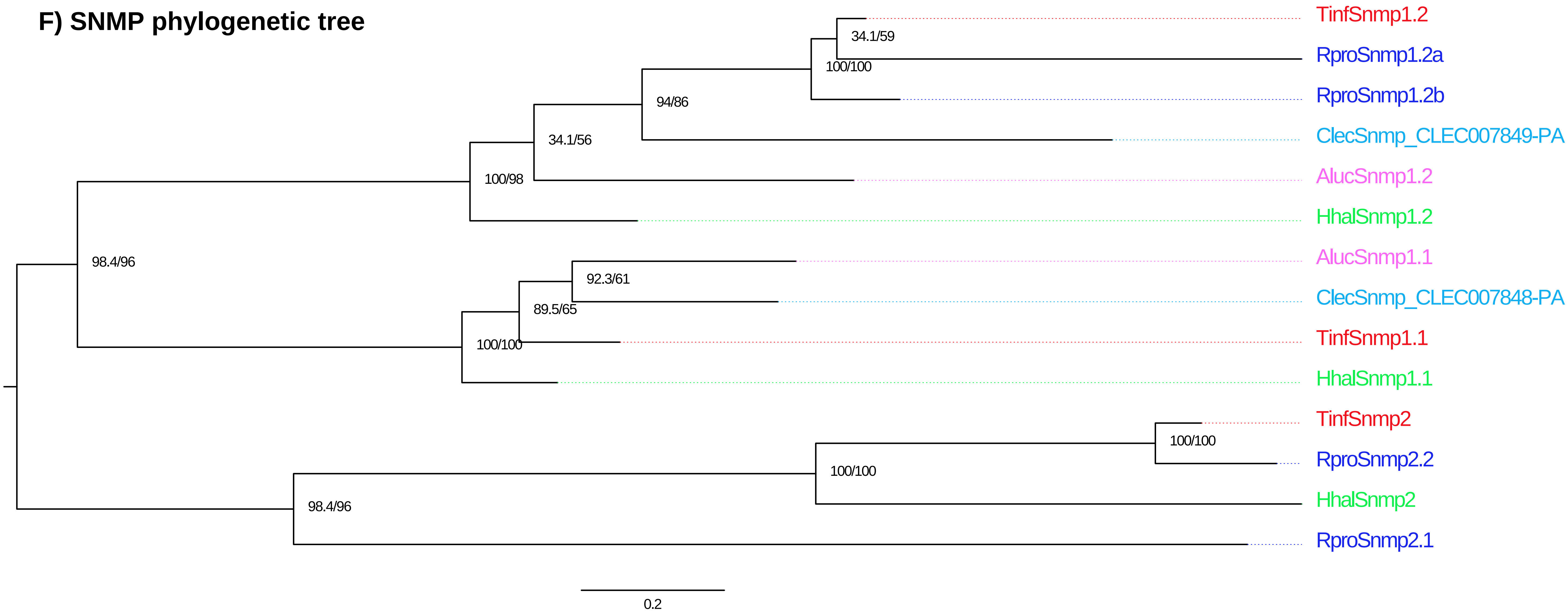

G) CheA and CheB phylogenetic tree

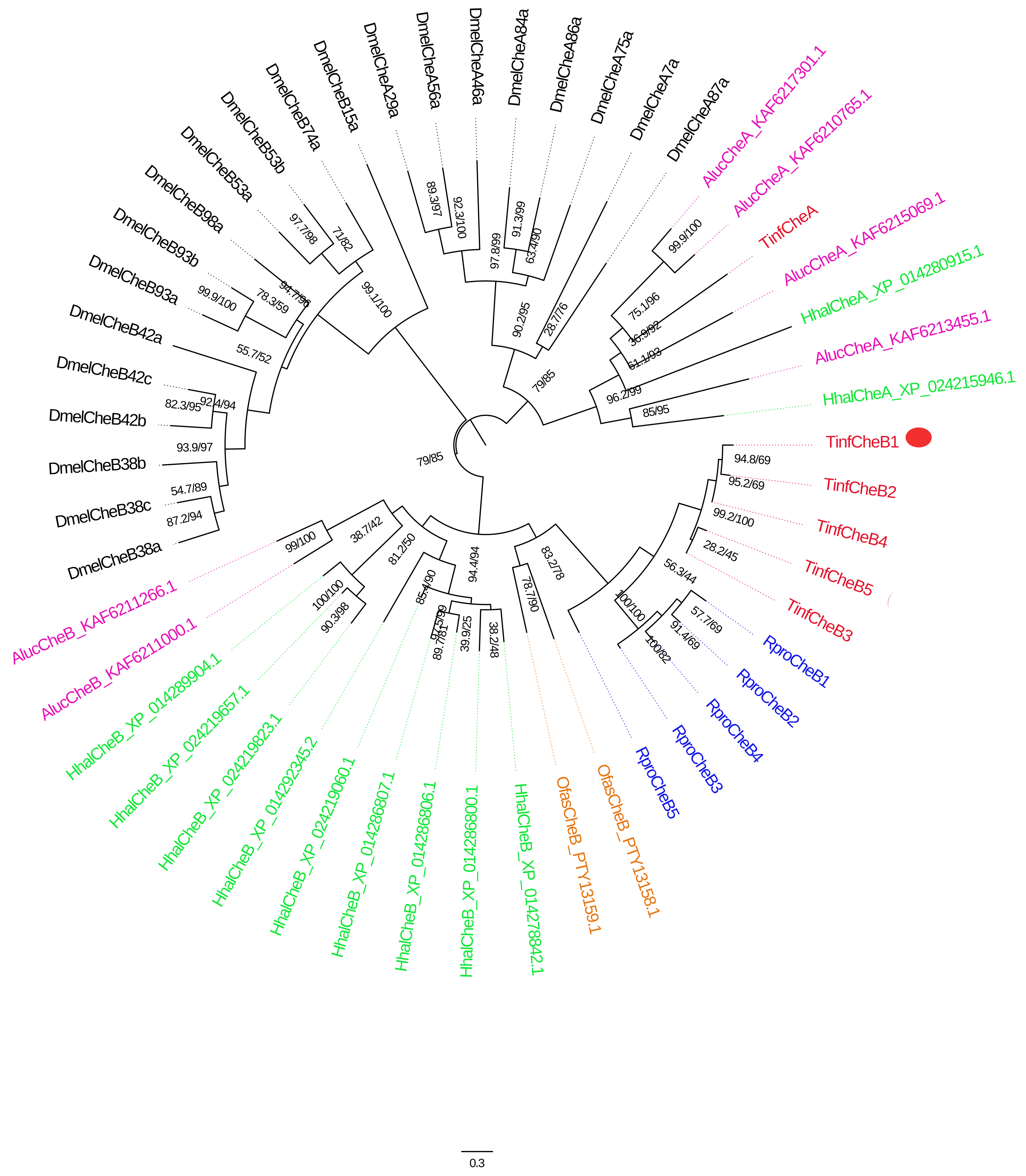

Supplement: Supplementary file 6 — Additional file 6: Supplementary Figure S2. Phylogenetic trees of sensory-related gene families analyzed. The maximum-likelihood trees were constructed using IQ-Tree and support values are shown for branches with both aLRT-SH and UFBoot values. Down and up-regulated transcripts after blood ingestion are marked with blue and red circles in the trees, respectively. The OR phylogenetic tree (A) was rooted using Orco and OR expansions that are exclusive to triatomines are highlighted in green. The GR phylogenetic tree (B) was rooted at the midpoint. The TO (C) phylogenetic tree was rooted at the midpoint. Cimex lectularius and O. fasciatus maintained their VectorBase and NCBI codes, respectively. In the case of T. dimidiata and T. pallidipennis, sequences were identified using the contig IDs from databases where their CDSs were obtained. Takeout sequences kept their assembly codes (if we were able to find them in their database). The TRP phylogenetic tree (D) was rooted at the midpoint. Apolygus lucorum, C. lectularius and O. fasciatus TRPs maintained their NCBI and VectorBase codes, while D. melanogaster sequences were obtained from Matsuara et al. (2009) [52]. The PPK phylogenetic tree (E) was rooted using Dmelppk17. Apolygus lucorum, C. lectularius and O. fasciatus PPKs maintained their NCBI and VectorBase codes, while D. melanogaster sequences were obtained from Zelle et al. (2013) [110]. The CHE phylogenetic tree (F) was rooted at the midpoint. Apolygus lucorum and O. fasciatus CheAs maintained their NCBI codes and D. melanogaster CheA and CheB sequences were obtained from Torres-Oliva et al. (2016) [111]. The SNMP phylogenetic tree (G) was rooted at the midpoint. Aluc - Apolygus lucorum, Clec - Cimex lectularius, Dmel - Drosophila melanogaster, Hhal - Halyomorpha halys, Ofas - Oncopeltus fasciatus, Rpro - Rhodnius prolixus, Tbra - Triatoma brasiliensis, Tdim - Triatoma dimidiata, Tinf - Triatoma infestans and Tpal - Triatoma pallidipennis. Sequences with TRINITY I [file 12864_2022_9059_MOESM6_ESM.pdf]
